# Supplementary material for: Transcriptional Differences of Coding and Non-Coding Genes Related to the Absence of Melanocyte in Skins of Bama Pig
Source: Genes (Basel). 2019 Dec 30;11(1):47. doi: 10.3390/genes11010047 (PMC7017308; doi:10.3390/genes11010047)
Supplement: Supplementary file 1 [file genes-11-00047-s001.zip › manuscript.docx]

*Article*

**Transcriptional differences of coding and non-coding genes related to the absence of melanocyte in skins of Bama pig**

**Long Jin ^1,†^, Lirui Zhao ^1,†^, Silu Hu ^1,†^, Keren Long ^1^, Pengliang Liu ^1^, Rui Liu ^1^, Xuan Zhou ^1^, Yixin Wang ^1^, Zhiqing Huang ^2^, Xuxu Lin ^1^, Qianzi Tang ^1,^* and Mingzhou Li ^1,^***

^1^ Farm Animal Genetic Resource Exploration and Innovation Key Laboratory of Sichuan Province, Sichuan Agricultural University, Chengdu, Sichuan 611130, China; longjin8806@163.com (L.J.); zlr779052043@gmail.com (L.Z.); erichu121@foxmail.com (S.H.); longkeren@163.com (K.L.); pengliangliu1995@163.com (P.L.); Lrui677@163.com (R.L.); zhouxuan198866@163.com (X.Z.); 444647336@qq.com (Y.W.); 297773480@qq.com (X.L.)

^2^ Key Laboratory for Animal Disease-Resistance Nutrition of China Ministry of Education, Institute of Animal Nutrition, Sichuan Agricultural University, Chengdu, Sichuan 611130, China; zqhuang@sicau.edu.cn (Z.H.)

***** Corresponding author. E-mail addresses: tangqianzi@sicau.edu.cn (Q.T.); mingzhou.li@sicau.edu.cn (M.L.); +86-180-1147-7512 (Q.T.); +86-133-4887-0312 (M.L.)

† These authors contributed equally to this work.

**Abstract:** Skin is the body’s largest organ, the main function of which is to protect underlying organs from possible external damages. Melanocyte plays an important role in skin pigmentation. Bama pig has “two-end-black” phenotype with different coat colors across skin regions, e.g. white skin (without melanocytes) and black skin (with melanocytes), which could be a model to investigate skin-related disorders, specifically loss of melanocytes. Here, we generated expression profiles of mRNAs and long noncoding RNAs in Bama pig skins with different coat colors. In total, 14,900 mRNAs and 7,549 lncRNAs were expressed. Overall, 2,338 mRNAs were identified as being differentially expressed, including 1,305 that were down-regulated and 1,033 up-regulated in white skin (*P* < 0.05; |log_2_(fold change)| > 1). The genes down-regulated in white skin were associated with pigmentation, melanocyte-keratinocyte interaction, and keratin, while up-regulated ones were mainly associated with cellular energy metabolisms. We also identified 113 differentially expressed lncRNAs. These lncRNAs may be implicated in pigmentation, keratin synthesis and cellar energy metabolism. These results suggested that the loss of melanocyte-keratinocyte interaction in white skin of Bama pigs could lead to possible distinct physiological properties, such as development of hypertrophic scar. In general, this study provides insight into the transcriptional difference involved in melanocyte-loss induced keratinocyte changes and promotes pig as biomedical models in skin research.

**Keywords:** melanocyte deficiency; Bama pig; model; transcriptome;

**1. Introduction**

Skin is the largest organ for mammals, exhibiting a complex heterogeneous and multilayered structure, and containing various components and more than 10 types of cells [[1](#_ENREF_1)]. This organ is the main barrier to the external environment and protects underlying organs from trauma and radiation damage.

Numerous researches have been performed to investigate or demonstrate the structural components and physiological mechanisms of skin in human and mouse. For example, pigmentation, of which the procedure depends on the production of eumelanin and pheomelanin in melanocytes, provides protections for the skin of humans and other animals, as well as directly affects their appearance [[2](#_ENREF_2)]. Researchers also demonstrated that the interaction of plasma membrane between melanocyte and keratinocyte is critically important during melanosome translation [[3](#_ENREF_3),[4](#_ENREF_4)].

Bama pig is an indigenous breed in China, with a “two-end-black” coat color phenotype (black head and hip, white belt across the body), and the white skin of Bama pig lacks melanocyte [[5](#_ENREF_5)]. With this particular character and the similarities of structure, biochemistry, immunology, molecular biology and clinical behavior in skin, Bama pig has been used for researches on wound healing and hypertrophic scar [[6](#_ENREF_6)], and also could be appropriate models to study the loss of melanocytes and diseases associated with melanocyte deficiency, such as Waardenburg syndrome (deafness and skin pigmentation deficiency) and vitiligo (skin pigmentation deficiency) [[7-9](#_ENREF_7)]. But still, the physiological mechanism of melanocyte, keratinocyte and other types of cells in black and white skin of Bama pig is not well understood due to few studies that were conducted on Bama pigs.

To expound biological mechanisms and interaction between melanocyte and other types of cells, we investigated the mRNA and lncRNA expression profiles of black and white skin in six Bama pigs. In the present study, we identified 2,338 (DE mRNAs) differentially expressed mRNAs and 113 differentially expressed lncRNAs (DE lncRNAs). The DE mRNAs were mainly associated with coat color, keratin, the TCA cycle and oxidative phosphorylation. As a result of functional enrichment analysis of lncRNA, we found four DE lncRNAs which might have potential roles in pigmentation, keratin synthesis and cellar energy metabolism. The melanocyte-keratinocyte interaction would loss as a result of melanocyte’s absent in white skin, even, the melanocyte’s deficiency could lead to possible distinct physiological properties, such as the development of hypertrophic scar.

2. Materials and Methods

2.1. Pig skin sampling

Six two-year-old female Bama pigs of a similar-weight and were raised under the same feeding and rearing conditions were chosen for the experiments. The pigs were humanely killed, after being stunned by an electric shock to ameliorate their suffering. Then, white skin from the back and black skin from the buttocks with 8 mm[[10](#_ENREF_10)] in-depth were collected (**Figure 1**). Each collected sample was put into a 1.5-ml tube with 1 ml of RNAlater (Life Technologies, Beijing, China). Moreover, eight tissues of three Bama pigs (heart, brain, liver, spleen, mesentery, longissimus dorsi, kidney, lung) were also collected. Finally, the tubes and tissues were immersed in liquid nitrogen and stored at −80℃. All experimental and sample collection procedures were approved by the Institutional Animal Care and Use Committee (IACUC) of the College of Animal Science and Technology of Sichuan Agricultural University, Sichuan, China, under permit No. DKY-S20163629.


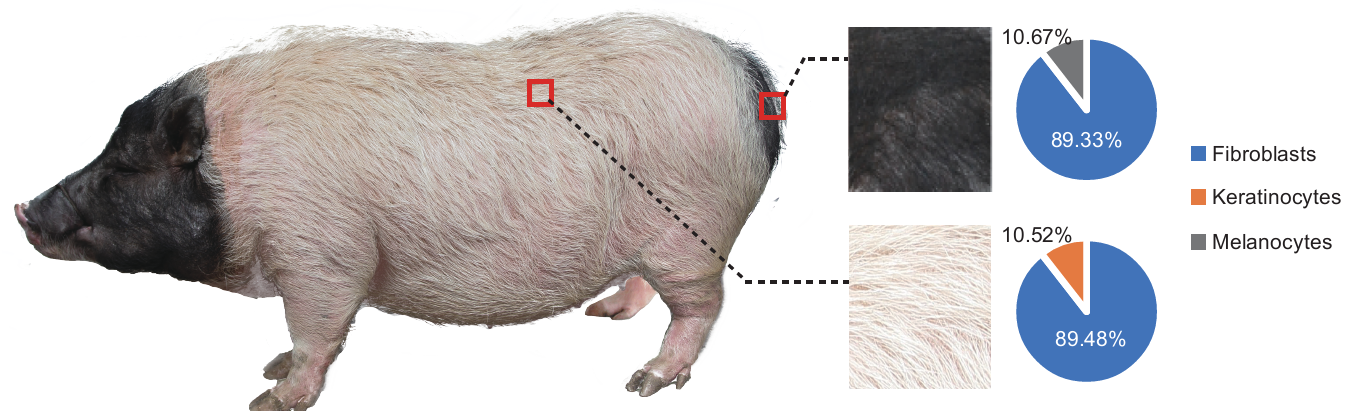


**Figure 1**. White and black skin were separately sampled from the pigs’ back and buttocks, and the pie plot shows the comparisons of Fibroblast, Keratinocyte and Melanocyte in two different skins after evaluated by CIBERSORT [[11](#_ENREF_11)].

2.2. Total RNA extraction, sequencing, and read mapping

A total of 3 μg of RNA (per skin sample) was extracted using Trizol reagent (Life Technologies, Beijing, China), in accordance with the manufacturer’s instructions, as was RNA of eight tissues from 3 Bama pigs (heart, brain, liver, spleen, mesentery, longissimus dorsi, kidney, lung). The integrity of RNA was checked by gel electrophoresis on 1.0% agarose gel with GoldView staining using an Agilent 2100 bioanalyzer. A NanoDrop spectrophotometer (Thermo Scientific) was used to measure the RNA concentrations. The RNAs with a ratio of absorbance at 260/280 nm of over 1.8 were selected for further study.

Six samples from three different pigs (named B1, B2, B3, W1, W2, and W3) were selected for sequencing. Approximately 1 µg of total RNA (per sample) and oligo(dT) magnetic heads were used for enriching poly(A) RNAs. The resulting fragments were used as a template for reverse transcription. Random hexamer primers, buffer, dNTPs, DNA polymerase I, and RNase H were used for generating RNA-Seq complementary DNA (cDNA) libraries; next, RNA-seq was conducted following the manufacturer’s standard procedures. High-quality strand-specific libraries were sequenced on the HiSeq X platform (Illumina, San Diego, CA, USA) and the bases were called using the software CASAVA v.1.8.2 (Illumina); then, 150-bp paired-end reads were obtained.

High-quality data were obtained by removing poly-N and low-quality reads from the raw data. As a consequence, a total of 44 Gb of clean data was acquired. The Q_30_ scores and GC content of the clean data were calculated. Clean data were mapped to the pig genome (*Sus scrofa* 11.1 from Ensembl) using TopHat (version 2.1.0) [[12](#_ENREF_12)]. (**Table 1**)

The RNA-Seq data have been deposited in NCBI’s (National Center for Biotechnology Information) Gene Expression Omnibus (GEO) and the accession number was GSE125517.

Table 1 Summary of data information.

| **Sample Name** | **Replicates** | **Raw Reads(M**^c^**)** | **Clean Reads(M)** | **Mapped Reads(M)** | **Mapped Ratio(**%**)** | **Proportion of Q**_30_ **(%)** |
| --- | --- | --- | --- | --- | --- | --- |
|  |  |  |  |  |  |  |
| W^a^ | 1 | 45.04 | 44.84 | 43.27 | 96.51 | 92.20 |
|  | 2 | 42.20 | 42.00 | 40.43 | 96.27 | 91.78 |
|  | 3 | 49.60 | 49.38 | 47.36 | 95.92 | 92.11 |
| B^b^ | 1 | 58.18 | 57.88 | 54.64 | 94.39 | 91.17 |
|  | 2 | 52.80 | 52.55 | 50.44 | 95.99 | 92.21 |
|  | 3 | 46.38 | 46.21 | 44.46 | 96.21 | 92.39 |

^a^: W represents the group of white skin samples of Bama pig;

^b^: B represents the Blacks;

^c^: M (Million Reads)

2.3. Messenger RNA analysis

The mRNA expression level of fragments per kilobase per million mapped reads (FPKM) for each sample was calculated with StringTie (version 1.3.3) [[13](#_ENREF_13)]; mRNAs with FPKM > 0.5 in at least one sample in at least one group were considered to be expressed. Then Cuffdiff (part of Cufflinks, version 2.2.1) [[12](#_ENREF_12)] was applied to detect differentially expressed genes, and those genes with adjusted-*P* values < 0.05 and |log_2_(FC)| > 1 were identified to be differentially expressed genes (DEGs).

2.4. Long noncoding RNAs’ identification

Mapped reads were assembled by StringTie and then merged with Cuffmerge (part of Cufflinks version 2.2.1) [[14](#_ENREF_14)]. Then, coding transcripts were filtered by the following steps: (1) remove transcripts of coding gene while comparing to annotated genome by Cuffcompare (part of Cufflinks) ; (2) comparing with the Pfam-27 database and trimming out transcripts with a *P*-value <10^−4^ by Hmmscan [[15](#_ENREF_15)]; (3) comparison with uniref and the nr database and trimming out transcripts with a *P*-value <10^−10^ by BLASTX (https://blast.ncbi.nlm.nih.gov/); and (4) prediction and calculation of the coding potential of the remaining transcripts by CPC [[16](#_ENREF_16)]. Transcripts without coding potential were retained. Finally, the expression levels of lncRNAs were calculated using StringTie. DE (differentially expressed) lncRNAs were detected by Cuffdiff. The lncRNAs with *P* values <0.05 were deemed to be differentially expressed (DE).

2.5. Clustering and Principal component analysis

FPKM values of the six samples (B1, B2, B3, W1, W2, and W3) were used for Principal component analysis (PCA) analysis as well as clustering analysis.

2.6. Profiling melanocyte proportion with CIBERSORT

The RNA-seq data of melanocyte, keratinocyte and fibroblasts were downloaded from reference[[17](#_ENREF_17)]. The CIBERSORT [[11](#_ENREF_11)] was used to estimate the proportion of melanocyte, keratinocyte and fibroblasts within skin.

2.7. Construction of lncRNA-mRNA interaction network

The R package WGCNA was used to detect interactions between lncRNA and mRNA[[18](#_ENREF_18)]. The Cytoscape (version 3.2.1)[[19](#_ENREF_19)] was used to construct the lncRNA-mRNA interaction network.

2.8. Functional enrichment analysis

Gene Ontology (GO) functional enrichment analysis and KEGG pathway functional enrichment analysis were performed with DE genes at the DAVID web server (<http://david.abcc.ncifcrf.gov/>). To predict the functions of the DE lncRNAs, the differentially expressed genes that were within 100 kb of the lncRNAs or showed a high correlation with the lncRNA were collected and performed functional enrichment analysis as well. The KEGG pathways or GO terms with Benjamini-corrected *P-*value < 0.05 were considered to be significant.

Pearson’s correlation coefficients between DE lncRNAs and the DE mRNAs were calculated with *Hmisc* (an R package from <https://cran.r-project.org/>), with the aim of identifying correlations between functional lncRNAs and DE mRNAs [[20](#_ENREF_20)].

2.9. Validation of genes and lncRNAs by Real-Time PCR

Twenty-one genes (3 lncRNA included) (**Table S1**) were selected for the validation and another eight tissues of three Bama pigs were used to investigate the regulations between the lncRNA *TCONS_00019024* and that of *CYGB* (n=3). To investigate the correlation between *TCONS_00019024* and *CYGB*, RNAs from the skin of six Bama pigs were used for the experiment (n=6). The genomic DNA in RNA samples was removed by gDNA Eraser (TaKaRa, Shanghai, China) at 42°C for 5 min. Five micrograms of RNA was reverse-transcribed into cDNA using RT Reagent Kit (TaKaRa, Shanghai, China). The primers for the genes and lncRNAs were designed using Primer 5.0 and tested by NCBI Primer-Blast. The volume of the reaction mixture was 10 µl, with 1 µl of cDNA, 0.5 µl of primers, 5 µl of SYBR (TaKaRa, Shanghai, China), and 3 µl of RNA-free water. The following RT-PCR reaction was performed for all genes and lncRNAs: 95°C for 3 min; followed by 40 cycles of 95°C for 10 s and amplification at the optimal temperature for each sample for 30 s; 95°C for 30 s; and then a melting curve analysis (65°C to 95°C). The expression of β-actin was used to correct the gene expression data. The 2^−ΔΔCT^ method was used to analyze the RT-PCR data and calculate relative expression. If a gene was up-regulated in black skin, its expression relative to that in white skin was calculated. If a gene was up-regulated in white skin, its expression relative to that in black skin was also calculated. The *t*-test was used to test the significance of differences in gene expression.

3. Results

3.1. Expression profiles of mRNAs and lncRNAs

Approximately 49.03 million raw reads were generated for each sample, and 43–58 million clean reads were obtained for further analysis. A total of 93%–97% of the clean reads can be mapped to the pig genome (**Table 1**). In total, 14,900 mRNAs and 7,549 lncRNA were identified to be expressed in our samples (**Tables S2** and **S3**), and the characteristic of lncRNA and mRNA transcripts showed that lncRNAs have fewer exon number (median value = 2), shorter transcript length (median value = 588nt), lower expression level (average FPKM value = 1.76) and lower coding potential, compared to mRNA (median of exon number = 8; median of transcript length = 2622nt; average FPKM value=30.72) which were consistent with previous researches (**Figure S1 A-D**) [[21-23](#_ENREF_21)]. Principal component analysis (PCA) (**Figure 2 A, B**), hierarchical cluster of expressed (**Figure S2 A, B**) or differentially expressed (**Figure S3 A, B**) mRNAs and lncRNA, as well as Pearson matrix correlation (**Figure S2 C, D**) based on expressed mRNA and lncRNA all indicated obvious variations between white and black skins. As shown in **Figure 1**, the fibroblast is the major cell type in skin, and the lack of melanocyte in the white skin, which was consistent with previous observation[[5](#_ENREF_5)].


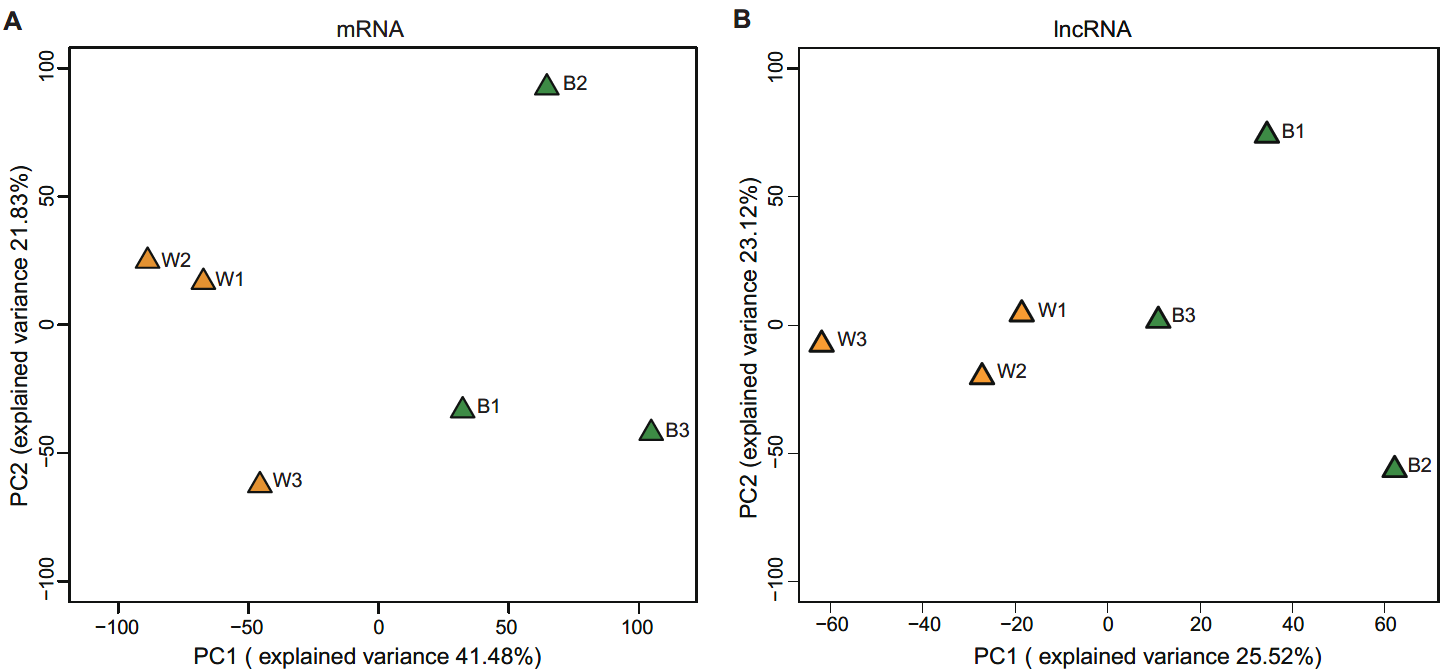


**Figure 2**. Principal component analysis of mRNA **(A)** and lncRNA **(B)**.

3.2. Functional enrichment analysis of DE mRNA

2,338 mRNAs were identified to be differentially expressed between two groups (**Table S4**). Among these DE mRNAs, 1,305 were down-regulated and 1,033 up-regulated in white skin (**Figure 3A**), including 239 genes down-regulated less than 0.25-fold and 295 genes up-regulated more than 4-fold (marked in **Table S4**).


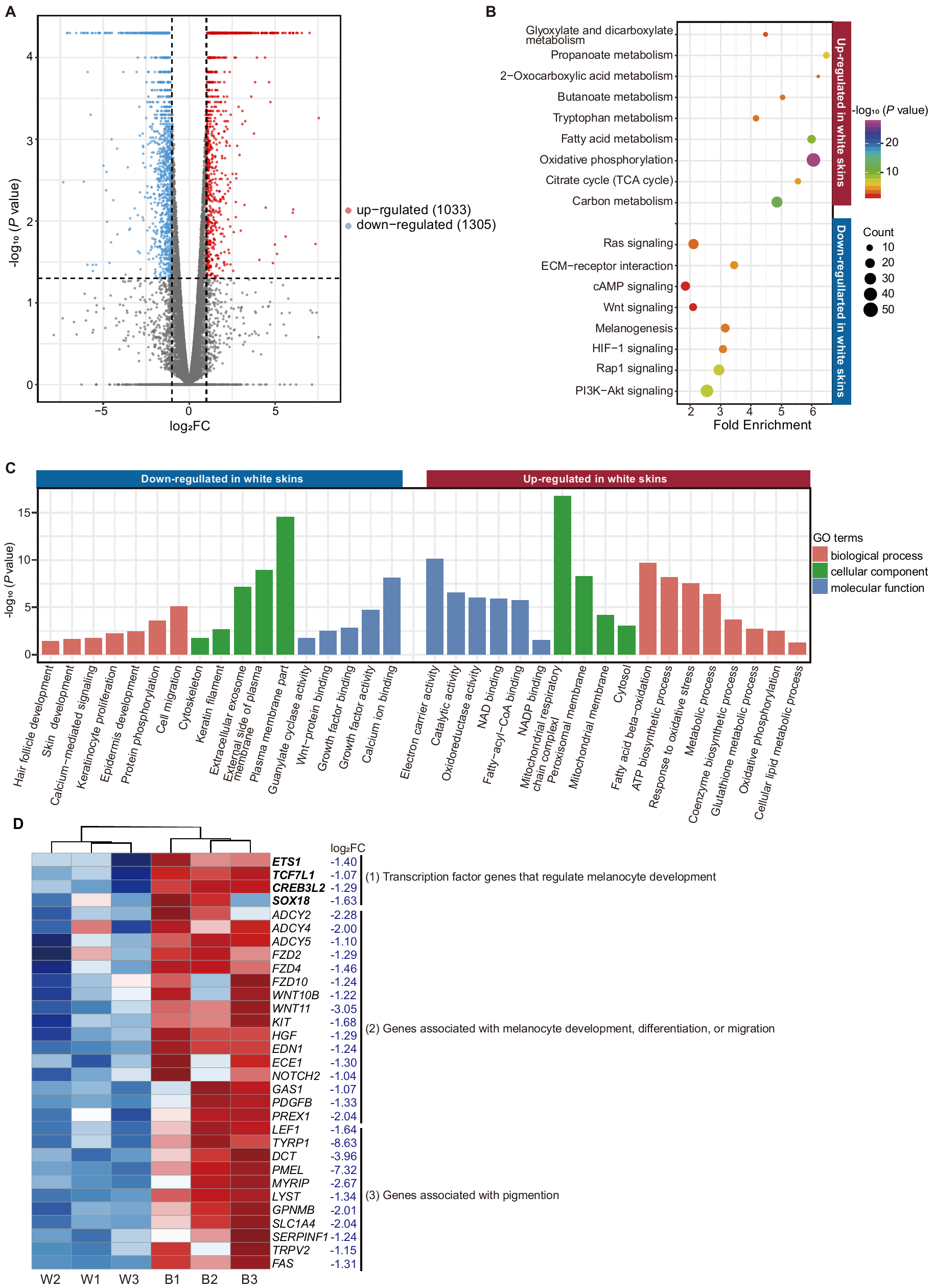


**Figure 3**. Enrichment analysis of DE mRNAs. **(A)** Volcano plot for 2,338 DE genes, of which, 1,305 were down-regulated in white skins, while 1,033 were up-regulated. **(B)** Functional enrichment analysis of the KEGG pathway (genes down-regulated in white skin were marked blue and up-regulated marked red; Benjamini corrected *P*-value < 0.05). **(C)** Gene Ontology (GO) enrichment analysis (genes down-regulated in white skin marked blue and up-regulated marked red *P*-value < 0.05). (**D)** Identification of genes associated with melanogenesis. The gene with bold are transcript factor genes (cluster (1)).

The down-regulated genes in white skin mainly related to coat color, such as, melanogenesis (*P* = 4.77 ×10^-4^), WNT signaling pathway (*P* = 9.86 ×10^-3^), PI3K-Akt signaling pathway (*P* = 7.49 ×10^-9^), cAMP signaling pathway (*P* = 9.53 ×10^-3^), and ECM-receptor interaction (*P* = 2.51×10^-5^) (**Figure 3B**, **Table S5**) [[24-29](#_ENREF_24)]. In addition, the well-known melanocyte-specific genes *TYR*, *TRPM1*, *TYRP1*, *PMEL* and *MLANA* were hardly expressed in white skin [[30](#_ENREF_30)](**Table 2**).

Table 2 Expression of the melanocyte-specific genes

| Gene Name | Up/down-regulated in white | FC | *P*-value | Functions |
| --- | --- | --- | --- | --- |
| *TYR* | Down | Un-expressed in white | 5.0 × 10^-5^ | Melanin synthesis[[2](#_ENREF_2)] |
| *TYRP1* | Down | B/W = 388:1 | 3.3 × 10^-2^ | Melanosomal protein [[2](#_ENREF_2)] |
| *TRPM1* | Down | B/W = 235:1 | 2.4 × 10^-1^ | Regulate tyrosinase activity [[31](#_ENREF_31)] |
| *MLANA* | Down | B/W = 164:1 | 1.5 × 10^-1^ | regulating *PMEL* processing [[32](#_ENREF_32)] |
| *PMEL (sliver*) | Down | B/W = 157:1 | 3.5 × 10^-3^ | Melanosome complex [[33](#_ENREF_33)] |
| *DCT* | Down | B/W = 15:1 | 5.0 × 10^-5^ | Melanosomal protein [[2](#_ENREF_2)] |

We also noticed 31 coat color genes (**Figure 3D**) down-regulated in white skin (*P* < 0.05; log_2_FC < −1), which were proved to be lowly expressed in white skin. [[33-40](#_ENREF_33)] Those 31 coat color genes could be divided into three classes: (1) transcription factor genes that regulate melanocyte development, such as *ETS1*, *TCF7L1*, and *SOX18* [[35](#_ENREF_35),[36](#_ENREF_36)]. (2) genes that regulate melanocyte development, differentiation, or migration, such as *KIT*, *HGF*, *EDN1* and *WNT11* [[2](#_ENREF_2),[7](#_ENREF_7),[41](#_ENREF_41),[42](#_ENREF_42)]. (3) genes associated with pigmentation, such as *DCT*, *TYRP1*, *PMEL* and *MYRIP* [[7](#_ENREF_7),[33](#_ENREF_33),[40](#_ENREF_40),[43](#_ENREF_43)]. Melanocytes produce melanin and secrete melanosomes, playing an irreplaceable role in pigmentation. Proteins encoded by genes in class (1) and (2) are required for normal development and the migration of melanocytes [[2](#_ENREF_2),[7](#_ENREF_7)]. Disruption of these genes would contribute to a lack of melanocytes in hair, skin, and the inner ear, and result in pigmentation deficiency [[7](#_ENREF_7),[35](#_ENREF_35),[38](#_ENREF_38),[41](#_ENREF_41),[44](#_ENREF_44)]. But for genes in class (3), the distribution of melanocyte was not affected by these genes’ decreasion[[2](#_ENREF_2),[7](#_ENREF_7)]. For example, *SOX* transcript factor family is required for melanocyte development, mutation of SOX gene family could result in Waardenburg syndrome [[45](#_ENREF_45)].*KIT* (c-kit) (log_2_FC = -1.68; *P* = 2.5 × 10^−4^) plays crucial roles in melanocyte development, melanocyte differentiation, and melanocyte migration [[2](#_ENREF_2),[7](#_ENREF_7),[46](#_ENREF_46)]. Mutation of *KIT* gene may cause melanocyte defective in European domestic pigs [[44](#_ENREF_44)]. While mutation of *TYRP1* or *PMEL* caused defective in eumelanin synthesis instead of melanocyte loss [[43](#_ENREF_43),[47](#_ENREF_47)]. Therefore, the genes in class (1) or (2) may play important roles in shaping the “two-end-black” phenotype. We also found these genes that may be implicated in pigmentation were down-regulated in white skin, such as keratins (*KRT*) gene [[48-50](#_ENREF_48)].

Except for pigmentation, we found numerous genes down-regulated in white skin were significantly enriched in subcategories close associated with the melanocyte-keratinocyte interaction, such as plasma membrane (384 genes) (*P* = 2.99 × 10^-15^), external side of the plasma membrane (41 genes) (*P* = 1.19 × 10^-9^), calcium ion binding (89 genes) (*P* = 7.60 × 10^-13^), and extracellular exosome (251 genes) (*P* = 7.33 × 10^-8^) (**Table S5**) as well as some terms closely related to skin’s structure like: keratin filament (*P* = 2.07 × 10^−3^), hair follicle development (P = 3.64 × 10-2) and skin development (*P* = 2.21 × 10^-2^) (**Table S5**) (**Figure 3C**).

While, up-regulated genes in white skin were mainly enriched in pathways link with cellular energy metabolism, such as oxidative phosphorylation (*P* = 6.78 × 10^-28^), carbon metabolism (*P* = 6.51 × 10^-13^), TCA cycle (*P* = 1.33 × 10^-5^), and propanoate metabolism (*P* = 7.37 × 10^-7^). (**Figure 3B**, **Table S5**). These observations suggest active cellular energy metabolism occurs in white skin compared with that in black skin. Besides, up-regulated gene were also significantly enriched in terms including catalytic activity (*P* = 2.87 × 10^-7^), oxidoreductase activity (*P* = 1.04 × 10^-6^), NADP binding (*P* = 3.04 × 10^-3^), fatty acid metabolic process (*P* = 5.11× 10^-5^), and mitochondrial membrane (*P* = 8.70 × 10^-4^) (for details, see **Table S5**) (**Figure 3C**).

3.3. Functional enrichment analysis of DE lncRNAs

113 lncRNA were identified to be differentially expressed, among which 34 were down-regulated and 79 were up-regulated in white skin (**Table S6**, **Figure 4A**). 88 DE mRNAs were oriented nearby those DE lncRNAs with 100kb. These DE mRNAs were found to be enriched in *PI3K*-*Alt* signaling pathway (*P* = 4.0 × 10^-2^) which were involved in pigmentation (**Figure 4B**) [[26](#_ENREF_26)].

In addition, we found four DE lncRNAs (*TCONS_00077733*, *TCONS_00042201*, *TCONS_00060772* and *TCONS_00019024*) might play roles in keratin synthesis, response to insulin, beta-oxidation of fat and melanocyte survival. For example, there were four keratin genes (*KRT80*, *KRT7*, *KRT81*, and *KRT86*) oriented nearby *TCONS_00077733* within 100kb, particularly *KRT80* which is convergent and 17,754 bp downstream of *TCONS_00077733* (r = 0.97, *P* = 7.60 × 10^−4^), which assumed that *TCONS_00077733* might be related to keratin synthesis. Moreover, *TCONS_00042201* is located sense with *IRS1* and 336 bp upstream of it (r = 0.85, *P* = 3.27 × 10^−2^). *IRS1* encodes insulin receptor substrate 1, which is associated with response to insulin [[51](#_ENREF_51)]. Mutation of IRS1 may thus contribute to insulin resistance [[51](#_ENREF_51)]. In addition, *TCONS_00060772* is an antisense lncRNA of *HSD17B4* and located overlaps with it (r = 0.99, *P* = 6.01 × 10^−6^). *HSD17B4* is involved in the beta-oxidation of fat [[52](#_ENREF_52)]. Besides, *TCONS_00019024* is divergent lncRNA [[21](#_ENREF_21)] of *CYGB* (cytoglobin), being located 3,888 bp upstream of *CYGB*. This lncRNA/mRNA pair was found to be up-regulated in black skin, showing a Pearson’s correlation coefficient of 0.932 (*P* = 1 × 10^−2^).(**Figure 4C**) *CYGB* is a *ROS* scavenger in melanocytes and plays a role in maintaining melanocyte survival [[53](#_ENREF_53)]. These results suggest some DE lncRNA may have a potential role in pigmentation, keratin synthesis and cellar energy metabolism.

Furtherly, 474 DE mRNAs showed high correlation (|r| > 0.95, *P* < 0.05) with 113 DE lncRNAs. These 474 DE mRNAs significantly enriched in Oxidative phosphorylation (*P* = 4.68 × 10^-8^), mitochondrial inner membrane (*P* = 6.46 × 10^-8^) and plasma membrane (*P* = 2.20 × 10^-2^) (**Figure 4D**). In addition, the lncRNA-mRNA interaction network indicated lncRNA *TCONS_00077733* may interact with *KRT* genes by trans (**Figure S4**). These results implied that DE lncRNA might be associated with cellar energy metabolism and melanocyte-keratinocyte interaction.


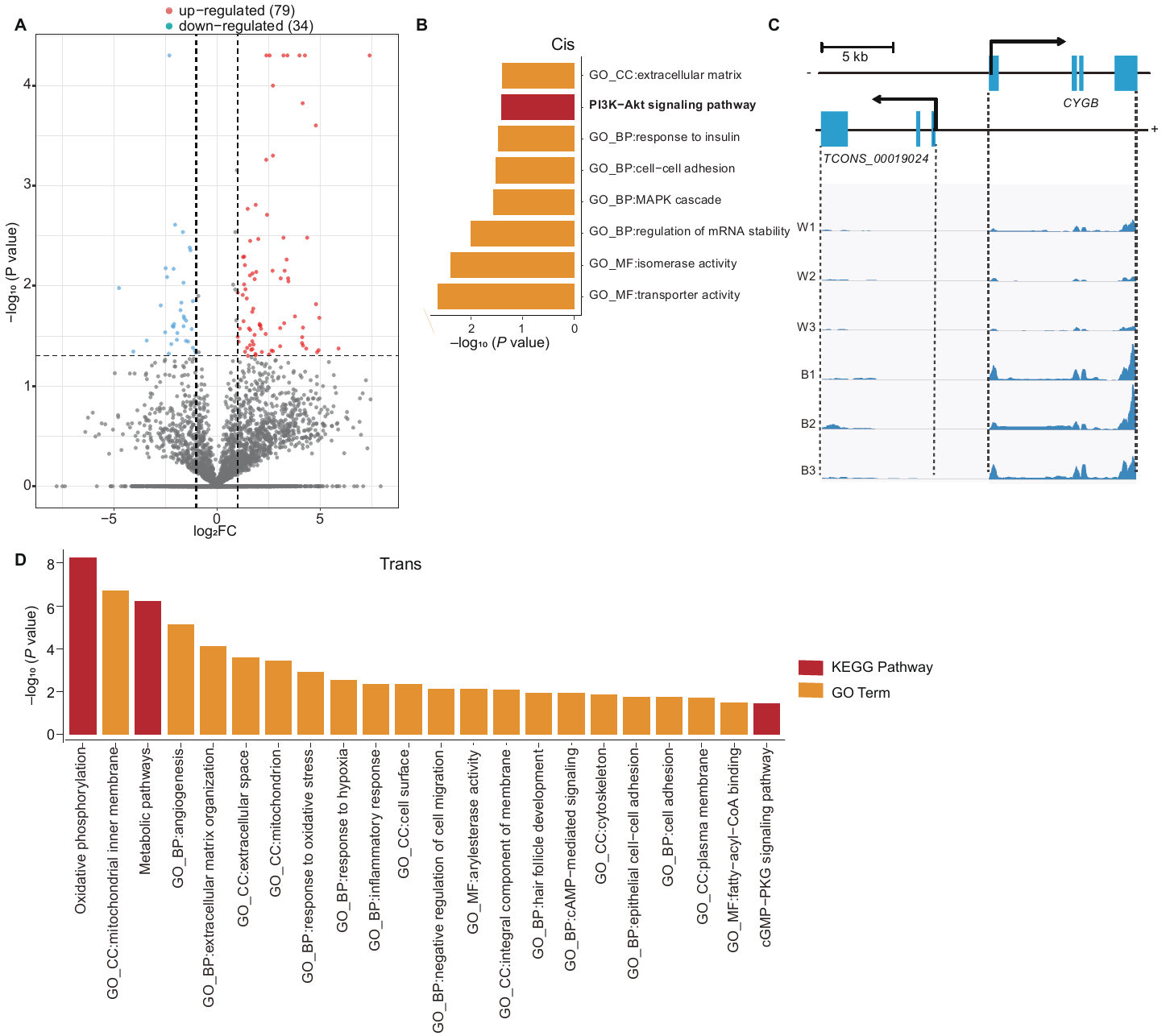


**Figure 4**. Functional enrichment analysis of DE lncRNAs. **(A)** DE lncRNAs between two groups (79 DE lncRNAs were up-regulated in white skin while 34 DE lncRNAs down-regulated). **(B)** Functional enrichment analysis with 88 DE genes adjacent to DE lncRNAs (*P*-value < 0.05). **(C)** Genomic location and reads abundance of *TCONS_00019024* and its target gene *CYGB*. (Pearson correlation coefficient: 0.932, P=0.01). **(D)** Functional enrichment analysis of trans target genes related to DE lncRNA.

3.4. Quantitative Real-Time PCR Validation

21 genes were selected for the validation. The melanocyte-specific genes (*TRPM1*, *TYRP1*, *PMEL*, *MLANA* and *DCT*) were significantly highly expressed in black skin and 21 genes above were indeed differentially expressed (**Figure 5A–C**).

RT-PCR indicated that the correlation coefficient between *TCONS_00019024* and *CYGB* was 0.71 in the skin (R^2^ = 0.50, *P* =3.5 × 10^−2^) (**Figure 5D**). We also investigated the relative expression of the TCONS_00019024/CYGB pair in eight other tissues. The results showed that *TCONS_00019024* and *CYGB* presented different expression patterns in the tissues, implying that the correlation of *TCONS_00019024* with *CYGB* may only occur in skin (**Figure 5E**). In conclusion, the qRT-PCR experiment validated well the discovery of RNA-Seq and supported the reliability of RNA-Seq.


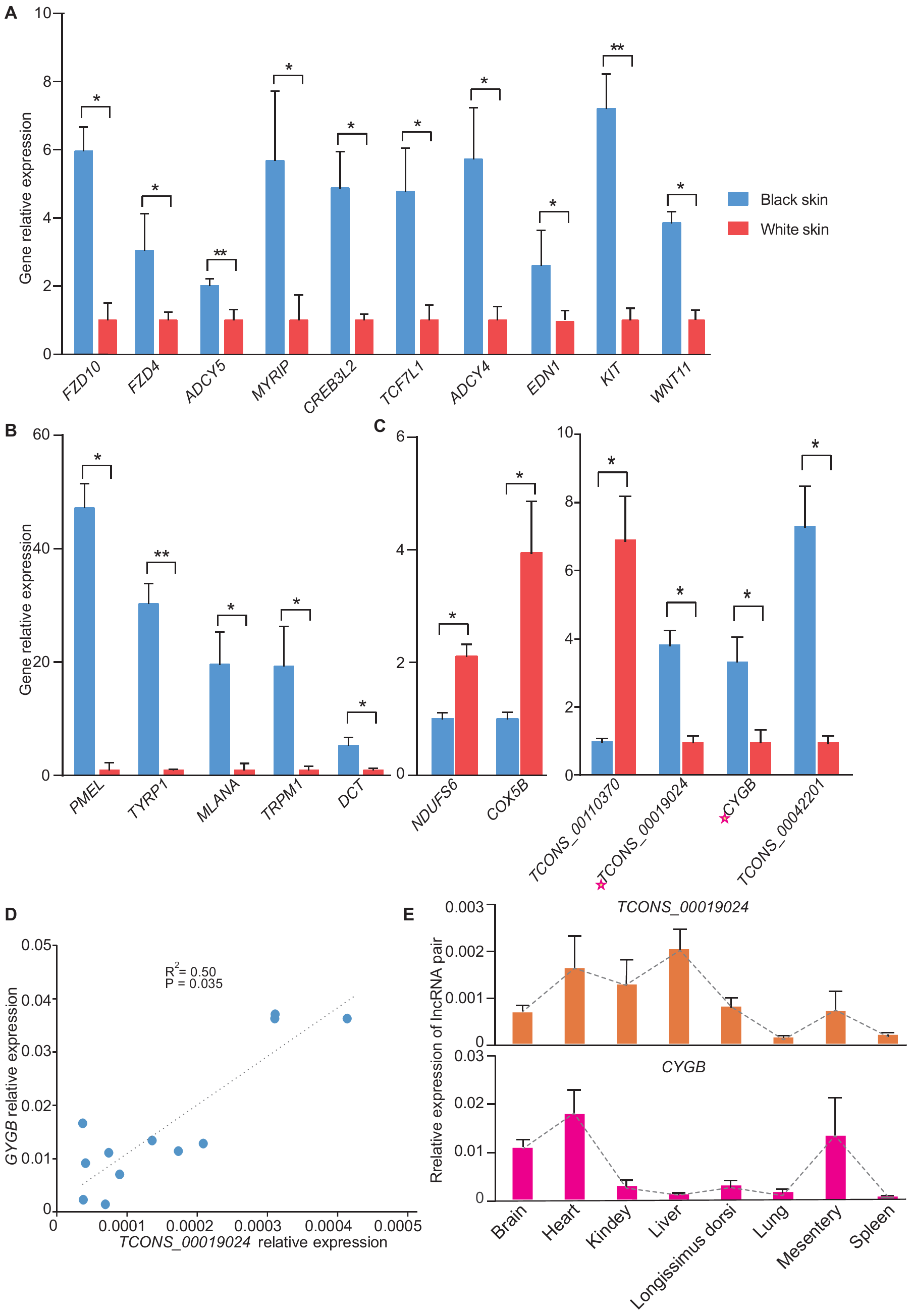


**Figure 5**. RT-PCR validation of the differentially expressed mRNAs and lncRNAs. **(A)** RT-PCR validation results of 10 coat color genes (n=3). **(B)** RT-PCR validation results of melanocyte-specific expression gene (n=3). **(C)** RT-PCR validation results of mRNA and lncRNA that differentially expressed in the skin (n=3). The lncRNA *TCONS_00019024* and its` target gene *CYGB* were marked utilizing pentagram. **(D)** Correlation curve of *TCONS_00019024*/*CYGB*. RT-PCR data from skins were used to create a correlation curve (n=6). Linear correlation coefficient of *TCONS_00019024*/*CYGB* were 0.71 (R^2^=0.50, P=0.035). **(E)** Relative expression of *TCONS_00019024* and *CYGB* in 8 tissues (n=3). * *P*-value < 0.05. ** *P*-value < 0.01.

4. Discussion and Conclusions

Skin is an important and the largest organ of the body, which provides protection for the underlying organs. Melanocyte inhabits in the skin whose major function is for the pigmentation of skin and hair. Loss of melanocyte would cause pigmentation deficiency. Mechanisms of melanocyte deficiency are still not fully understood and the transcriptome profiles study related to the lack of melanocytes are insufficient. Even the mechanism associated with the cross-talk between melanocyte and other types of cells in the skin is still not well understood in the pig. Therefore, we performed transcriptomic analysis of Bama pig skin to reveal the mechanism potentially associated with the loss of melanocytes or interaction networks related to melanocyte in the pig.

Previous research showed that the correlation coefficient of mRNA expression between the skin and other tissues was 0.7–0.9 in humans [[54](#_ENREF_54)], while such a correlation between white and black skins was 0.92 (**Figure S2 C-D**). These studies indicated that the similarity of gene expression level between white and black skin was greater than that between the skin and other tissues. Even with a higher similarity, the skin in the different regions of the body is distinct in skin thickness and PH[[55](#_ENREF_55),[56](#_ENREF_56)]. The present study showed that the skin in two parts may differ in thickness and PH as well as the appearance, cellular energy metabolism and with/without melanocyte-keratinocyte interaction. As is well-illustrated that melanin synthesis in melanocyte[[2](#_ENREF_2)]. It’s hard for white skin to synthesize melanin without melanocyte, and no pigmentations in white skin. Differential expression of coat color gene could reflect the discrepancy in melanin synthesis between the skin between black and white skin.

Melanocyte and keratinocyte are important compositions of skin and there is a close interaction between keratinocyte and melanocyte in the skin. The absence of melanocyte in white skin could result in the disappearance of melanocyte-keratinocyte interaction and may impact on the function of keratinocyte. In human, the calcium (Ca^2+^) plays an important role in the interaction of keratinocytes and melanocytes. Moreover, the interaction between the keratinocyte plasma membrane and melanocyte plasma membrane was shown to be essential for melanosome transfer [[3](#_ENREF_3),[4](#_ENREF_4)]. Interestingly, many down-regulated genes in white skin were associated with calcium release or the plasma membrane, such as *ITSN1* (log_2_FC = −1.72, *P* = 5 × 10^−5^), *BTK* (log_2_FC = −1.01, *P* = 4.72 × 10^−2^), *FYN* (log_2_FC = −1.32, *P* = 5 × 10^−5^), and *CCR*1 (log_2_FC = −1.69, *P* = 1.5 × 10^−4^) (**Figure 3C**, **Table S4**). Downregulation of these genes could provide evidence of the disappearance of melanocyte-keratinocyte interaction in the white skin of Bama pig. Moreover, keratinocyte plays an important role during development of hypertrophic scar [[57](#_ENREF_57)]. Melanocyte could accelerate wound closure by stimulating keratinocyte proliferation and development of scar by stimulating the growth and pro­liferation of fibroblasts[[58](#_ENREF_58),[59](#_ENREF_59)]. Comparison with white skins, the black skins owned more melanocyte. The black skin may be more prone to hypertrophic scars than the white skin. Previous wound healing experiments also demonstrated the skin of Duroc pig (with melanocyte) is more prone to hypertrophic scars than the skin of Hampshire pig (melanocyte deficiency)[[60-62](#_ENREF_60)]. Therefore, the black skin of the Bama pig may be more suitable for hypertrophic scar research.

In this study, we identified two DE lncRNAs (*TCONS_00077733*, and *TCONS_00060772*) which might be involved in keratin synthesis and cellar energy metabolism. The black skin was different from the white skin in the expression of keratin genes and genes related to cellular energy metabolism. lncRNA was evidenced to play critical roles in the skin, such as melanin synthesis for goat [[63](#_ENREF_63)], psoriasis for human[[23](#_ENREF_23)] and expression of keratin and promote melanogenesis in mouse[[64](#_ENREF_64)]. In addition, lncRNA *H19* regulates Dsg1 expression and the consequent keratinocyte differentiation through acting as an endogenous “sponge” of *miR-130b-3p* in human[[65](#_ENREF_65)]. Thus, we deduced that *TCONS_00077733* and *TCONS_00060772* might be one of cause responsible for the difference between black and white skin, more evidence is needed to support this assumption. Taken together, the results of this study provide evidence of the interactions involved in pigmentation. This research provides insight into the complex mechanisms associated with the “two-end-black” phenotype.

In conclusion, we systematically identified mRNAs and lncRNAs in the black and white skin of Bama pig. Our results suggested that the loss of melanocytes could contribute to the expression of melanogenesis genes, and lack of melanocytes might be the primary cause that the white skin differs from the black skin in the keratinocyte’s function and the development of hypertrophic scar. In addition, the present research implies that lncRNAs play roles in skin. Our research could promote the application of Bama pig in research on melanocyte deficiency.

**Supplementary Materials**: Figure S1: Characteristics’ comparison of mRNAs and lncRNAs in exon numbers (A), CDS (coding sequence) length (B), expression levels (C) and coding potential (D) (calculated by CPC2). Figure S2: Hierarchical cluster analysis of expressed mRNA (A) and lncRNA (B). Pairwise Pearson correlations of expressed mRNA (C) and lncRNA (D). Figure S3: Hierarchical cluster analysis of differentially expressed mRNA (A) and lncRNA (B). Figure S4: The view of lncRNA-mRNA associated network. Table S1: Primer sequences of selected genes. Table S2: Expressed mRNAs. Table S3: Expressed lncRNAs. Table S4: Differentially expressed mRNAs. Table S5: Functional enrichment analysis of DEGs up or down-regulated in white skin. Table S6: Differentially expressed lncRNAs. Table S7: Functional enrichment analysis of cis target genes. Table S8: Functional enrichment analysis of trans target genes.

**Founding:** This work was supported by grants from the National Key R&D Program of China ( 2018YFD0500403 and 2018YFD0501204), the National Natural Science Foundation of China (31601919, 31872335 and 31772576), the Sichuan Province & Chinese Academy of Science of Science & Technology Cooperation Project (2017JZ0025), the Key Project of Sichuan Education Department (17ZA0306), the Young Talent Lifting Project of Sichuan Association for Science and Technology (2018RCTJ).

**Author Contributions:** Conceptualization, Long Jin, Mingzhou Li and Qianzi Tang; Data curation, Silu Hu; Formal analysis, Long Jin, Lirui Zhao and Silu Hu; Funding acquisition, Mingzhou Li; Investigation, Keren Long, Rui Liu, Xuan Zhou, Yixin Wang, Zhiqing Huang and Xuxu Lin; Methodology, Qianzi Tang; Project administration, Mingzhou Li and Qianzi Tang; Validation, Pengliang Liu; Visualization, Long Jin and Lirui Zhao; Writing – original draft, Lirui Zhao and Silu Hu; Writing – review & editing, Long Jin.

**Conflict of interest:** The authors declare no conflict of interest.

Reference

1. Kanitakis, J. Anatomy, histology and immunohistochemistry of normal human skin. *European journal of dermatology : EJD* **2002**, *12*, 390-399; quiz 400-391.

2. Cichorek, M.; Wachulska, M.; Stasiewicz, A.; Tyminska, A. Skin melanocytes: Biology and development. *Postepy dermatologii i alergologii* **2013**, *30*, 30-41.

3. Joshi, P.G.; Nair, N.; Begum, G.; Joshi, N.B.; Sinkar, V.P.; Vora, S. Melanocyte-keratinocyte interaction induces calcium signalling and melanin transfer to keratinocytes. *Pigment cell research* **2007**, *20*, 380-384.

4. Bush, W.D.; Simon, J.D. Quantification of ca(2+) binding to melanin supports the hypothesis that melanosomes serve a functional role in regulating calcium homeostasis. *Pigment cell research* **2007**, *20*, 134-139.

5. Liu, Y.; Chen, J.Y.; Shang, H.T.; Liu, C.E.; Wang, Y.; Niu, R.; Wu, J.; Wei, H. Light microscopic, electron microscopic, and immunohistochemical comparison of bama minipig (sus scrofa domestica) and human skin. *Comp Med* **2010**, *60*, 142-148.

6. Liu, B.; Liu, Y.; Wang, L.; Hou, C.; An, M. Rna-seq-based analysis of the hypertrophic scarring with and without pressure therapy in a bama minipig model. *Scientific reports* **2018**, *8*, 11831.

7. Lin, J.Y.; Fisher, D.E. Melanocyte biology and skin pigmentation. *Nature* **2007**, *445*, 843-850.

8. Tachibana, M.; Kobayashi, Y.; Matsushima, Y. Mouse models for four types of waardenburg syndrome. *Pigment cell research* **2003**, *16*, 448-454.

9. Lerner, A.B.; Shiohara, T., .; Boissy, R.E.; Jacobson, K.A.; Lamoreux, M.L.; Moellmann, G.E. A mouse model for vitiligo. *Journal of Investigative Dermatology* **1986**, *87*, 299-304.

10. Fan, R.; Xie, J.; Bai, J.; Wang, H.; Tian, X.; Bai, R.; Jia, X.; Yang, L.; Song, Y.; Herrid, M.*, et al.* Skin transcriptome profiles associated with coat color in sheep. *BMC genomics* **2013**, *14*, 389.

11. Chen, B.; Khodadoust, M.S.; Liu, C.L.; Newman, A.M.; Alizadeh, A.A. Profiling tumor infiltrating immune cells with cibersort. *Methods in molecular biology* **2018**, *1711*, 243-259.

12. Ghosh, S.; Chan, C.K. Analysis of rna-seq data using tophat and cufflinks. *Methods in molecular biology* **2016**, *1374*, 339-361.

13. Pertea, M.; Pertea, G.M.; Antonescu, C.M.; Chang, T.C.; Mendell, J.T.; Salzberg, S.L. Stringtie enables improved reconstruction of a transcriptome from rna-seq reads. *Nature biotechnology* **2015**, *33*, 290-295.

14. Finn, R.D.; Bateman, A.; Clements, J.; Coggill, P.; Eberhardt, R.Y.; Eddy, S.R.; Heger, A.; Hetherington, K.; Holm, L.; Mistry, J.*, et al.* Pfam: The protein families database. *Nucleic acids research* **2014**, *42*, D222-230.

15. Finn, R.D.; Clements, J.; Arndt, W.; Miller, B.L.; Wheeler, T.J.; Schreiber, F.; Bateman, A.; Eddy, S.R. Hmmer web server: 2015 update. *Nucleic acids research* **2015**, *43*, W30-38.

16. Kong, L.; Zhang, Y.; Ye, Z.Q.; Liu, X.Q.; Zhao, S.Q.; Wei, L.; Gao, G. Cpc: Assess the protein-coding potential of transcripts using sequence features and support vector machine. *Nucleic acids research* **2007**, *35*, W345-349.

17. Reemann, P.; Reimann, E.; Ilmjärv, S.; Porosaar, O.; Silm, H.; Jaks, V.; Vasar, E.; Kingo, K.; Kõks, S. Melanocytes in the skin--comparative whole transcriptome analysis of main skin cell types. *PloS one* **2014**, *9*, e115717.

18. Langfelder, P.; Horvath, S. Wgcna: An r package for weighted correlation network analysis. *BMC Bioinformatics* **2008**, *9*, 559.

19. Shannon, P.; Markiel, A.; Ozier, O.; Baliga, N.S.; Wang, J.T.; Ramage, D.; Amin, N.; Schwikowski, B.; Ideker, T. Cytoscape: A software environment for integrated models of biomolecular interaction networks. *Genome research* **2003**, *13*, 2498-2504.

20. Jin, L.; Hu, S.; Tu, T.; Huang, Z.; Tang, Q.; Ma, J.; Wang, X.; Li, X.; Zhou, X.; Shuai, S.*, et al.* Global long noncoding rna and mrna expression changes between prenatal and neonatal lung tissue in pigs. *Genes* **2018**, *9*.

21. Luo, S.; Lu, J.Y.; Liu, L.; Yin, Y.; Chen, C.; Han, X.; Wu, B.; Xu, R.; Liu, W.; Yan, P.*, et al.* Divergent lncrnas regulate gene expression and lineage differentiation in pluripotent cells. *Cell stem cell* **2016**, *18*, 637-652.

22. Li, B.; Tsoi, L.C.; Swindell, W.R.; Gudjonsson, J.E.; Tejasvi, T.; Johnston, A.; Ding, J.; Stuart, P.E.; Xing, X.; Kochkodan, J.J.*, et al.* Transcriptome analysis of psoriasis in a large case-control sample: Rna-seq provides insights into disease mechanisms. *The Journal of investigative dermatology* **2014**, *134*, 1828-1838.

23. Tsoi, L.C.; Iyer, M.K.; Stuart, P.E.; Swindell, W.R.; Gudjonsson, J.E.; Tejasvi, T.; Sarkar, M.K.; Li, B.; Ding, J.; Voorhees, J.J.*, et al.* Analysis of long non-coding rnas highlights tissue-specific expression patterns and epigenetic profiles in normal and psoriatic skin. *Genome biology* **2015**, *16*, 24.

24. Jin, E.J.; Erickson, C.A.; Takada, S.; Burrus, L.W. Wnt and bmp signaling govern lineage segregation of melanocytes in the avian embryo. *Developmental biology* **2001**, *233*, 22-37.

25. Rabbani, P.; Takeo, M.; Chou, W.; Myung, P.; Bosenberg, M.; Chin, L.; Taketo, M.M.; Ito, M. Coordinated activation of wnt in epithelial and melanocyte stem cells initiates pigmented hair regeneration. *Cell* **2011**, *145*, 941-955.

26. Larribere, L.; Khaled, M.; Tartare-Deckert, S.; Busca, R.; Luciano, F.; Bille, K.; Valony, G.; Eychene, A.; Auberger, P.; Ortonne, J.P.*, et al.* Pi3k mediates protection against trail-induced apoptosis in primary human melanocytes. *Cell death and differentiation* **2004**, *11*, 1084-1091.

27. Rodriguez, C.I.; Setaluri, V. Cyclic amp (camp) signaling in melanocytes and melanoma. *Archives of biochemistry and biophysics* **2014**, *563*, 22-27.

28. D'Orazio, J.; Fisher, D.E. Central role for camp signaling in pigmentation and uv resistance. *Cell cycle* **2011**, *10*, 8-9.

29. Bhat, B.; Singh, A.; Iqbal, Z.; Kaushik, J.K.; Rao, A.R.; Ahmad, S.M.; Bhat, H.; Ayaz, A.; Sheikh, F.D.; Kalra, S.*, et al.* Comparative transcriptome analysis reveals the genetic basis of coat color variation in pashmina goat. *Scientific reports* **2019**, *9*, 6361.

30. Sheinboim, D.; Maza, I.; Dror, I.; Parikh, S.; Krupalnik, V.; Bell, R.E.; Zviran, A.; Suita, Y.; Hakim, O.; Mandel-Gutfreund, Y.*, et al.* Oct4 impedes cell fate redirection by the melanocyte lineage master regulator mitf in mouse escs. *Nature communications* **2017**, *8*, 1022.

31. Devi, S.; Kedlaya, R.; Maddodi, N.; Bhat, K.M.; Weber, C.S.; Valdivia, H.; Setaluri, V. Calcium homeostasis in human melanocytes: Role of transient receptor potential melastatin 1 (trpm1) and its regulation by ultraviolet light. *American journal of physiology. Cell physiology* **2009**, *297*, C679-687.

32. Toshihiko, H.; Hidenori, W.; Jacqueline, M.; Yuji, Y.; Vieira, W.D.; Hearing, V.J. Mart-1 is required for the function of the melanosomal matrix protein pmel17/gp100 and the maturation of melanosomes. *Journal of Biological Chemistry* **2005**, *280*, 14006-14016.

33. Hellstrom, A.R.; Watt, B.; Fard, S.S.; Tenza, D.; Mannstrom, P.; Narfstrom, K.; Ekesten, B.; Ito, S.; Wakamatsu, K.; Larsson, J.*, et al.* Inactivation of pmel alters melanosome shape but has only a subtle effect on visible pigmentation. *PLoS genetics* **2011**, *7*, e1002285.

34. Seberg, H.E.; Van Otterloo, E.; Loftus, S.K.; Liu, H.; Bonde, G.; Sompallae, R.; Gildea, D.E.; Santana, J.F.; Manak, J.R.; Pavan, W.J.*, et al.* Tfap2 paralogs regulate melanocyte differentiation in parallel with mitf. *PLoS genetics* **2017**, *13*, e1006636.

35. Saldana-Caboverde, A.; Perera, E.M.; Watkins-Chow, D.E.; Hansen, N.F.; Vemulapalli, M.; Mullikin, J.C.; Program, N.C.S.; Pavan, W.J.; Kos, L. The transcription factors ets1 and sox10 interact during murine melanocyte development. *Developmental biology* **2015**, *407*, 300-312.

36. Kawakami, A.; Fisher, D.E. The master role of microphthalmia-associated transcription factor in melanocyte and melanoma biology. *Laboratory investigation; a journal of technical methods and pathology* **2017**, *97*, 649-656.

37. Schouwey, K.; Delmas, V.; Larue, L.; Zimber-Strobl, U.; Strobl, L.J.; Radtke, F.; Beermann, F. Notch1 and notch2 receptors influence progressive hair graying in a dose-dependent manner. *Developmental dynamics : an official publication of the American Association of Anatomists* **2007**, *236*, 282-289.

38. Lindsay, C.R.; Lawn, S.; Campbell, A.D.; Faller, W.J.; Rambow, F.; Mort, R.L.; Timpson, P.; Li, A.; Cammareri, P.; Ridgway, R.A.*, et al.* P-rex1 is required for efficient melanoblast migration and melanoma metastasis. *Nature communications* **2011**, *2*, 555.

39. Park, P.J.; Lee, T.R.; Cho, E.G. Substance p stimulates endothelin 1 secretion via endothelin-converting enzyme 1 and promotes melanogenesis in human melanocytes. *The Journal of investigative dermatology* **2015**, *135*, 551-559.

40. Kuroda, T.S.; Mitsunori, F. Functional analysis of slac2-c/myrip as a linker protein between melanosomes and myosin viia. *Journal of Biological Chemistry* **2005**, *280*, 28015-28022.

41. Pillaiyar, T.; Manickam, M.; Jung, S.H. Recent development of signaling pathways inhibitors of melanogenesis. *Cellular signalling* **2017**, *40*, 99-115.

42. Takeo, M.; Lee, W.; Rabbani, P.; Sun, Q.; Hu, H.; Lim, C.H.; Manga, P.; Ito, M. Ednrb governs regenerative response of melanocyte stem cells by crosstalk with wnt signaling. *Cell reports* **2016**, *15*, 1291-1302.

43. Ren, J.; Mao, H.; Zhang, Z.; Xiao, S.; Ding, N.; Huang, L. A 6-bp deletion in the tyrp1 gene causes the brown colouration phenotype in chinese indigenous pigs. *Heredity* **2011**, *106*, 862-868.

44. Pielberg, G.; Olsson, C.; Syvanen, A.C.; Andersson, L. Unexpectedly high allelic diversity at the kit locus causing dominant white color in the domestic pig. *Genetics* **2002**, *160*, 305-311.

45. Southard-Smith, E.M.; Kos, L.; Pavan, W.J. Sox10 mutation disrupts neural crest development in dom hirschsprung mouse model. *Nature genetics* **1998**, *18*, 60-64.

46. Liao, C.P.; Booker, R.C.; Morrison, S.J.; Le, L.Q. Identification of hair shaft progenitors that create a niche for hair pigmentation. *Genes & development* **2017**, *31*, 744-756.

47. Andersson, L.S.; Wilbe, M.; Viluma, A.; Cothran, G.; Ekesten, B.; Ewart, S.; Lindgren, G. Equine multiple congenital ocular anomalies and silver coat colour result from the pleiotropic effects of mutant pmel. *PloS one* **2013**, *8*, e75639.

48. Irvine, A.D.; McKenna, K.E.; Jenkinson, H.; Hughes, A.E. A mutation in the v1 domain of keratin 5 causes epidermolysis bullosa simplex with mottled pigmentation. *The Journal of investigative dermatology* **1997**, *108*, 809-810.

49. Geller, L.; Kristal, L.; Morel, K.D. Epidermolysis bullosa simplex with mottled pigmentation due to a rare keratin 5 mutation: Cutaneous findings in infancy. *Pediatric dermatology* **2013**, *30*, 631-632.

50. Lo Cicero, A.; Delevoye, C.; Gilles-Marsens, F.; Loew, D.; Dingli, F.; Guere, C.; Andre, N.; Vie, K.; van Niel, G.; Raposo, G. Exosomes released by keratinocytes modulate melanocyte pigmentation. *Nature communications* **2015**, *6*, 7506.

51. Ryu, J.; Galan, A.K.; Xin, X.; Dong, F.; Abdul-Ghani, M.A.; Zhou, L.; Wang, C.; Li, C.; Holmes, B.M.; Sloane, L.B.*, et al.* Appl1 potentiates insulin sensitivity by facilitating the binding of irs1/2 to the insulin receptor. *Cell reports* **2014**, *7*, 1227-1238.

52. de Launoit, Y.; Adamski, J. Unique multifunctional hsd17b4 gene product: 17beta-hydroxysteroid dehydrogenase 4 and d-3-hydroxyacyl-coenzyme a dehydrogenase/hydratase involved in zellweger syndrome. *Journal of molecular endocrinology* **1999**, *22*, 227-240.

53. Fujita, Y.; Koinuma, S.; De Velasco, M.A.; Bolz, J.; Togashi, Y.; Terashima, M.; Hayashi, H.; Matsuo, T.; Nishio, K. Melanoma transition is frequently accompanied by a loss of cytoglobin expression in melanocytes: A novel expression site of cytoglobin. *PloS one* **2014**, *9*, e94772.

54. Fagerberg, L.; Hallstrã¶M, B.M.; Oksvold, P.; Kampf, C.; Djureinovic, D.; Odeberg, J.; Habuka, M.; Tahmasebpoor, S.; Danielsson, A.; Edlund, K. Analysis of the human tissue-specific expression by genome-wide integration of transcriptomics and antibody-based proteomics. *Molecular & Cellular Proteomics* **2014**, *13*, 397-406.

55. Lee, Y.; Hwang, K. Skin thickness of korean adults. *Surgical and radiologic anatomy : SRA* **2002**, *24*, 183-189.

56. Wilhelm, K.P.; Cua, A.B.; Maibach, H.I. Skin aging. Effect on transepidermal water loss, stratum corneum hydration, skin surface ph, and casual sebum content. *Archives of dermatology* **1991**, *127*, 1806-1809.

57. Ghahary, A.; Ghaffari, A. Role of keratinocyte-fibroblast cross-talk in development of hypertrophic scar. *Wound repair and regeneration : official publication of the Wound Healing Society [and] the European Tissue Repair Society* **2007**, *15 Suppl 1*, S46-53.

58. H-O, R.; Busche, M.N.; Knobloch, K., .; Tenenhaus, M., . Is uv radiation beneficial in postburn wound healing? *Medical Hypotheses* **2010**, *75*, 436-438.

59. Gao, F.L.; Jin, R.; Zhang, L.; Zhang, Y.G. The contribution of melanocytes to pathological scar formation during wound healing. *International journal of clinical and experimental medicine* **2013**, *6*, 609-613.

60. Gallant-Behm, C.L.; Tsao, H.; Reno, C.; Olson, M.E.; Hart, D.A. Skin wound healing in the first generation (f1) offspring of yorkshire and red duroc pigs: Evidence for genetic inheritance of wound phenotype. *Burns : journal of the International Society for Burn Injuries* **2006**, *32*, 180-193.

61. Johansson Moller, M.; Chaudhary, R.; Hellmen, E.; Hoyheim, B.; Chowdhary, B.; Andersson, L. Pigs with the dominant white coat color phenotype carry a duplication of the kit gene encoding the mast/stem cell growth factor receptor. *Mammalian genome : official journal of the International Mammalian Genome Society* **1996**, *7*, 822-830.

62. Gallant-Behm, C.L.; Hart, D.A. Genetic analysis of skin wound healing and scarring in a porcine model. *Wound repair and regeneration : official publication of the Wound Healing Society [and] the European Tissue Repair Society* **2006**, *14*, 46-54.

63. Ren, H.; Wang, G.; Chen, L.; Jiang, J.; Liu, L.; Li, N.; Zhao, J.; Sun, X.; Zhou, P. Genome-wide analysis of long non-coding rnas at early stage of skin pigmentation in goats (capra hircus). *BMC genomics* **2016**, *17*, 67.

64. Ji, K.; Fan, R.; Zhang, J.; Yang, S.; Dong, C. Long non-coding rna expression profile in cdk5-knockdown mouse skin. *Gene* **2018**, *672*, 195-201.

65. Li, C.X.; Li, H.G.; Huang, L.T.; Kong, Y.W.; Chen, F.Y.; Liang, J.Y.; Yu, H.; Yao, Z.R. H19 lncrna regulates keratinocyte differentiation by targeting mir-130b-3p. *Cell death & disease* **2017**, *8*, e3174.


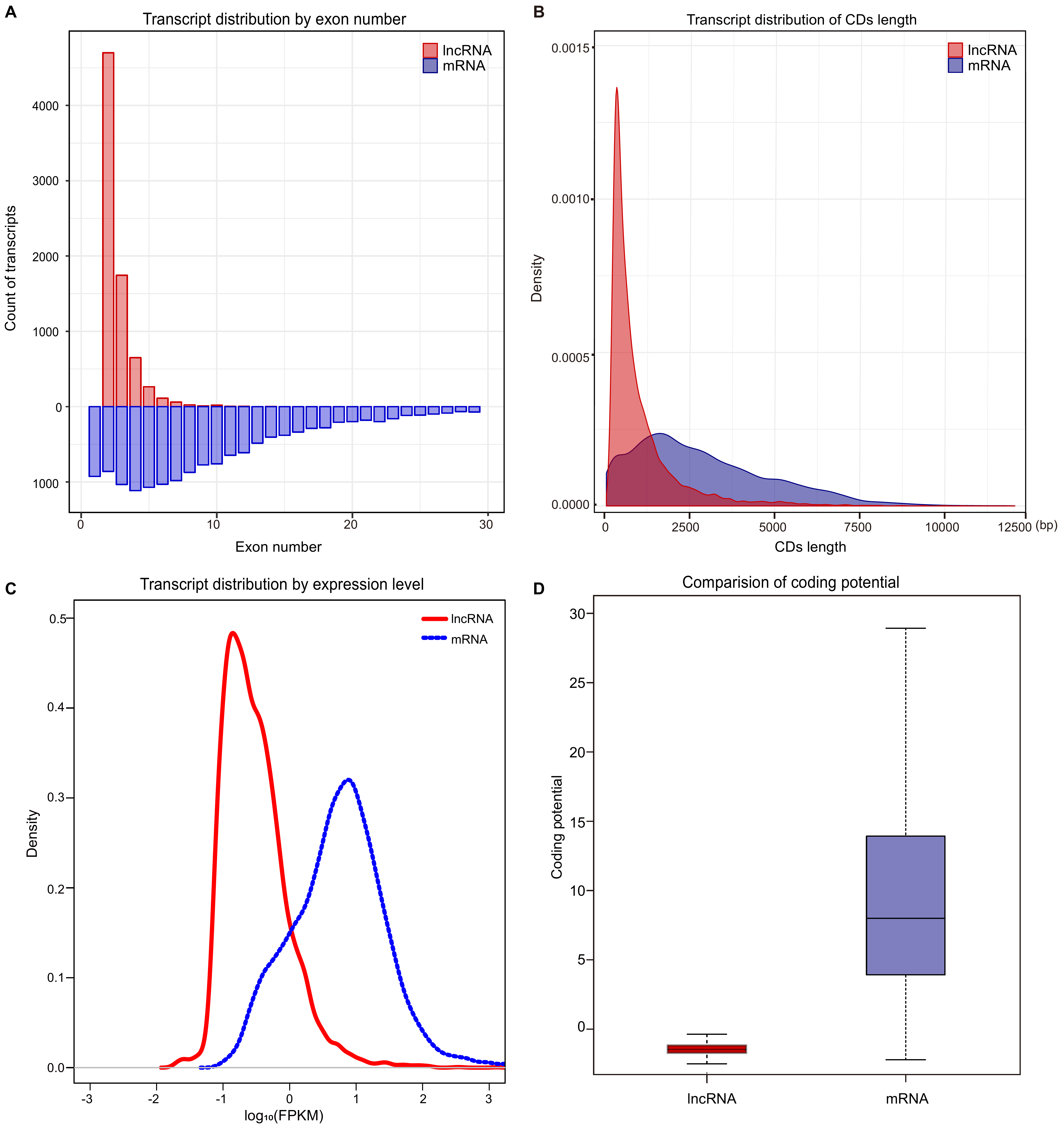


**Figure S1.** Characteristics’ comparison of mRNAs and lncRNAs in exon numbers **(A),** CDS (coding sequence) length **(B)**, expression levels **(C)** and coding potential **(D)** (calculated by CPC2).


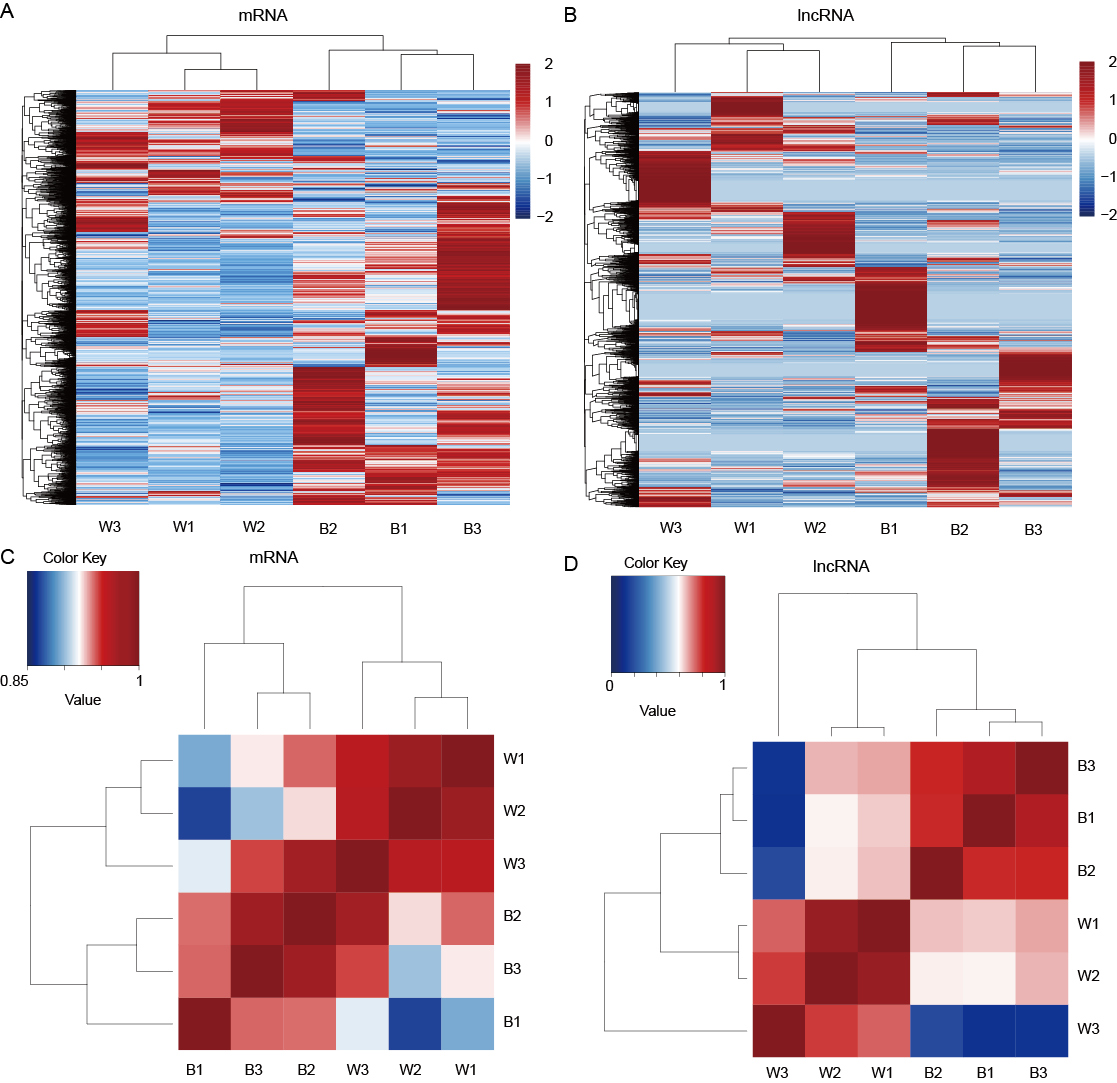


**Figure S2:** Hierarchical cluster analysis of expressed mRNA **(A)** and lncRNA **(B)**. Pairwise Pearson correlations of expressed mRNA **(C)** and lncRNA **(D)**.


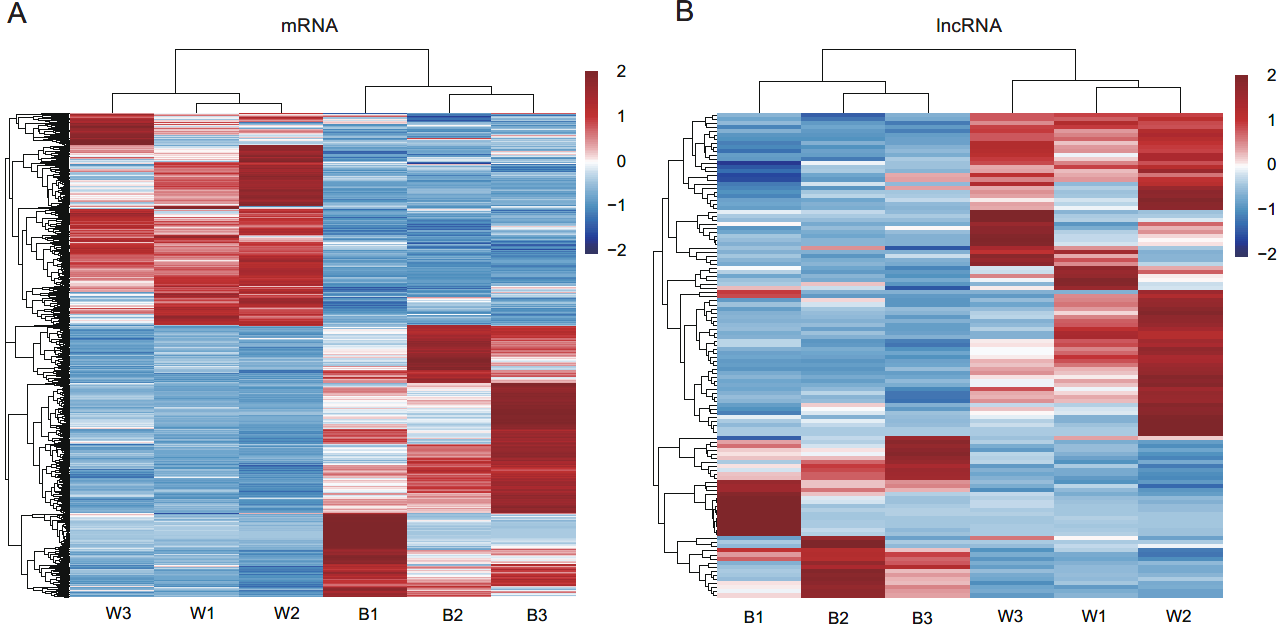


**Figure S3.** Hierarchical cluster analysis of differentially expressed mRNA **(A)** and lncRNA **(B)**.


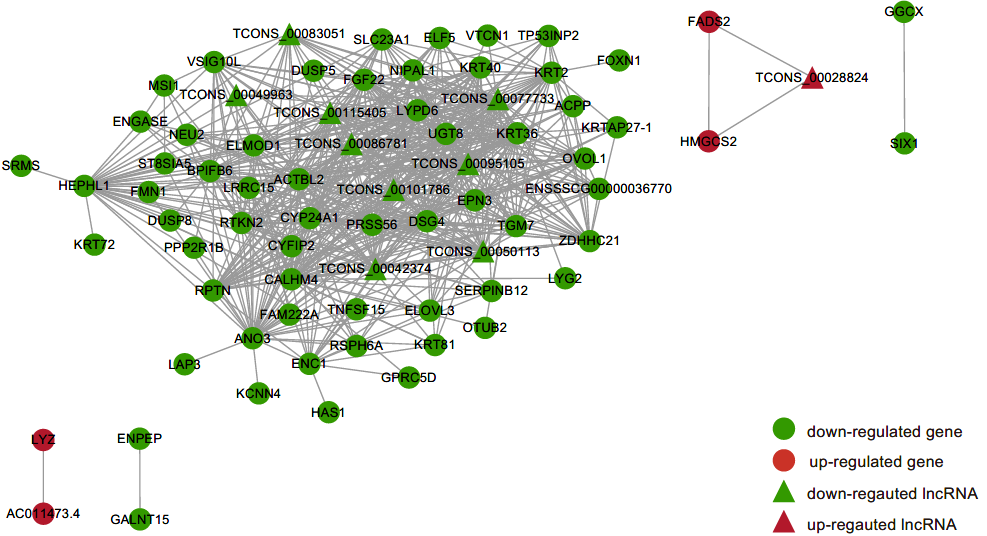


**Figure S4.** The view of lncRNA-mRNA associated network.
