# Supplementary figures and images for: Transcriptional Differences of Coding and Non-Coding Genes Related to the Absence of Melanocyte in Skins of Bama Pig
Source: Genes (Basel). 2019 Dec 30;11(1):47. doi: 10.3390/genes11010047 (PMC7017308; doi:10.3390/genes11010047)

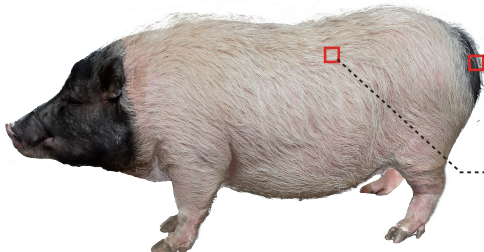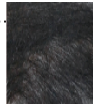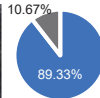

- Fibroblasts
- Keratinocytes
- Melanocytes

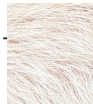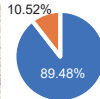

Supplement: Supplementary file 1 [file genes-11-00047-s001.zip › Figure.1.pdf]

**A**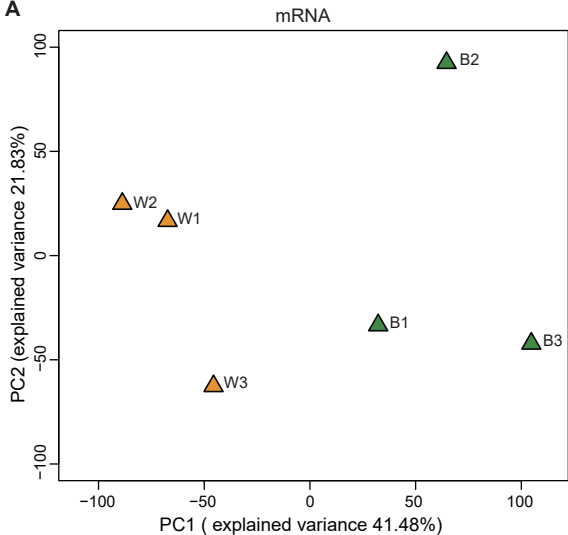**B**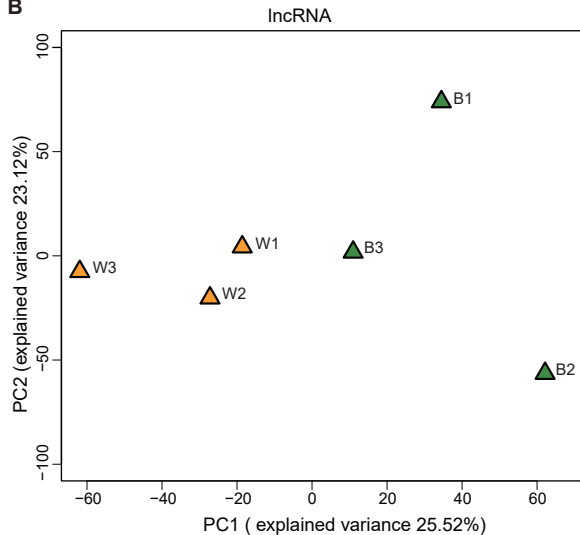

Supplement: Supplementary file 1 [file genes-11-00047-s001.zip › Figure.2.pdf]

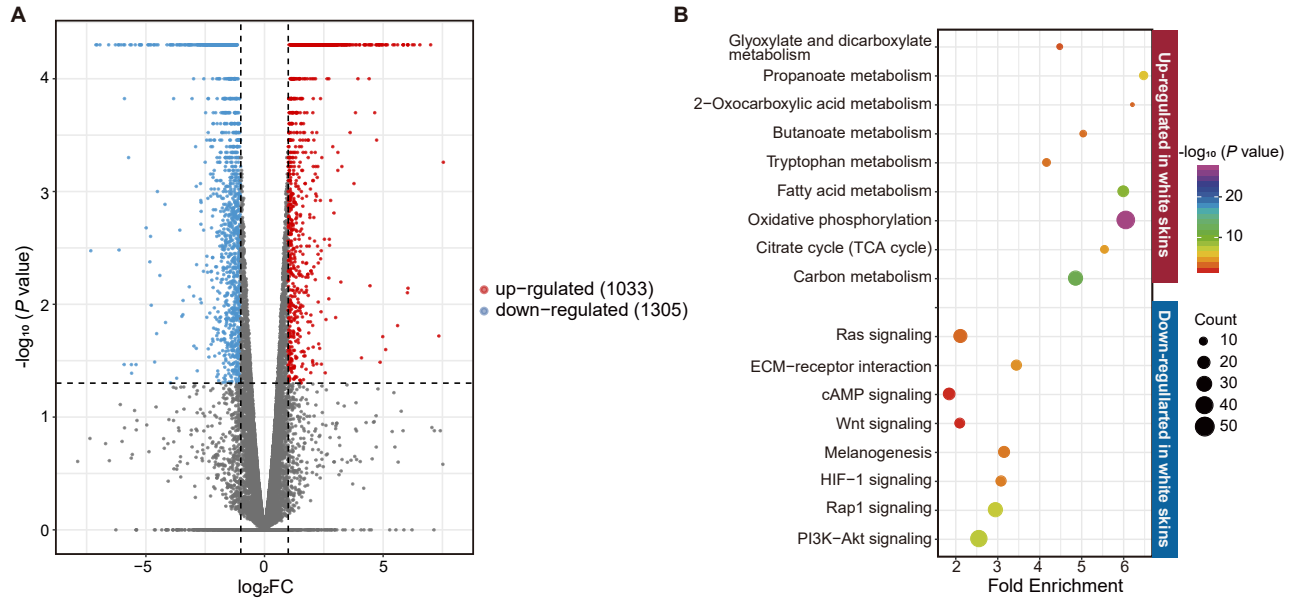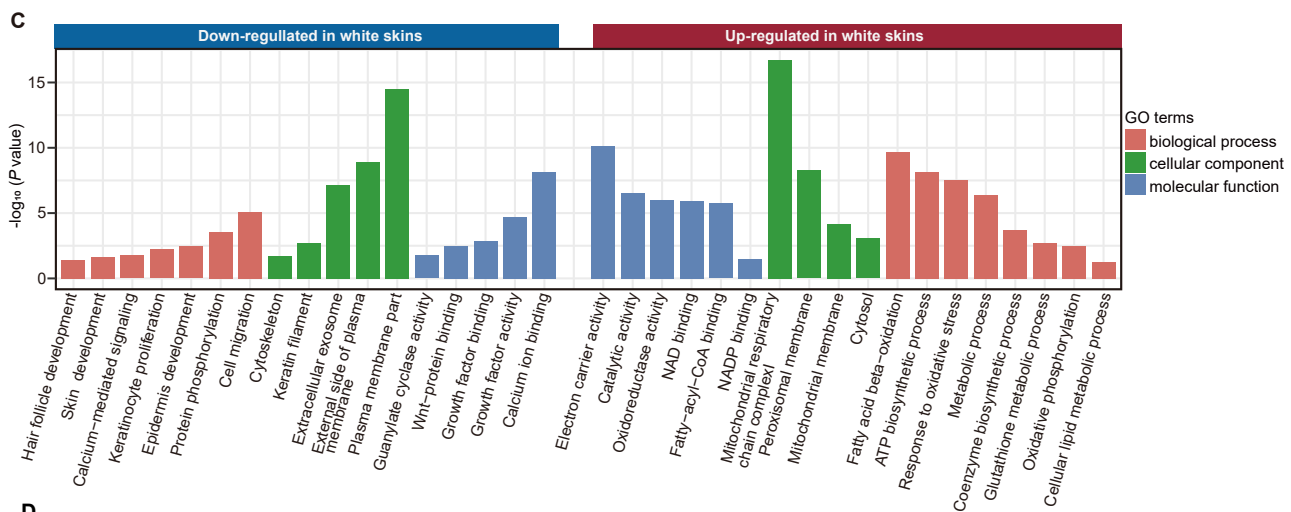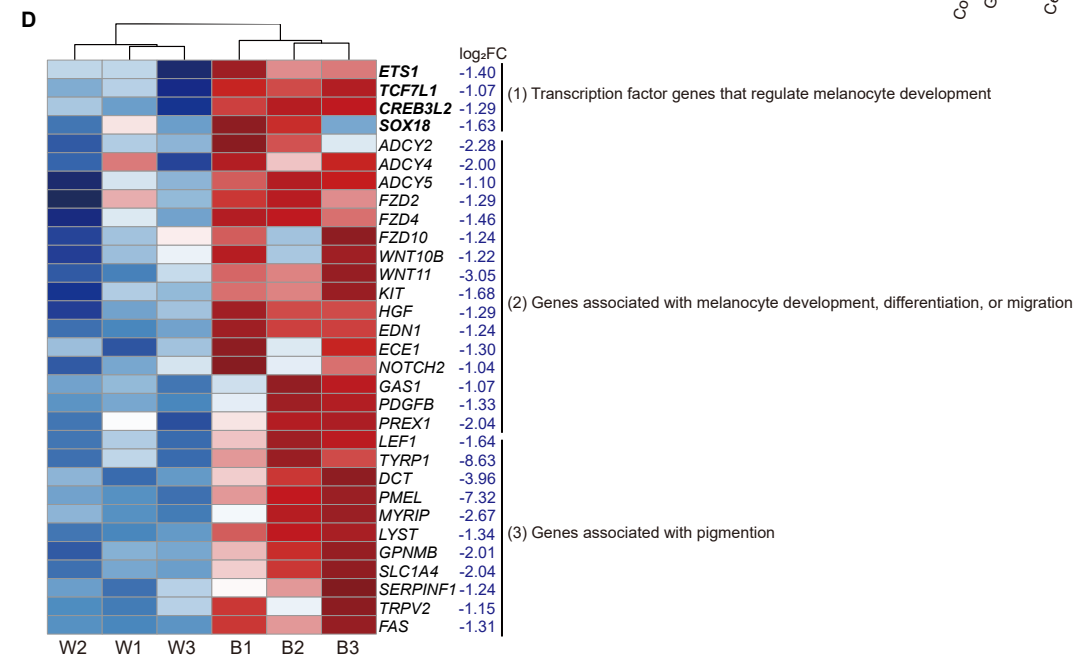

Supplement: Supplementary file 1 [file genes-11-00047-s001.zip › Figure.3.pdf]

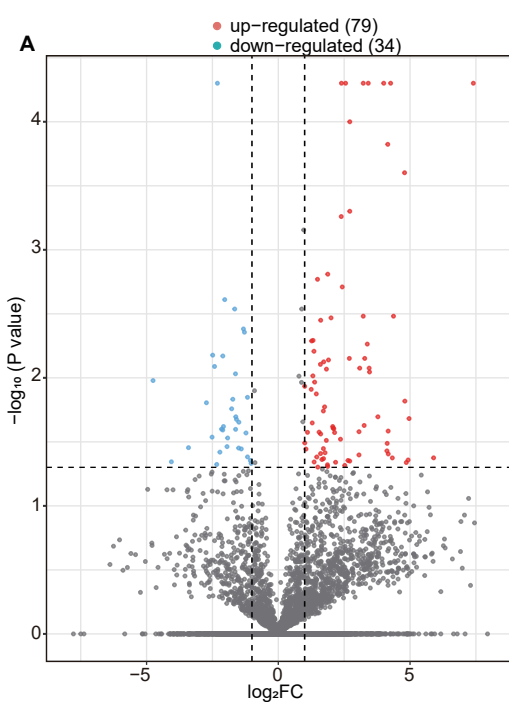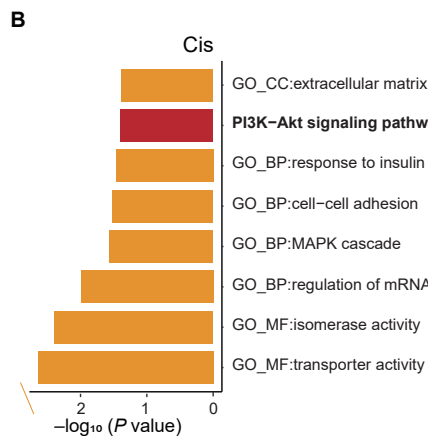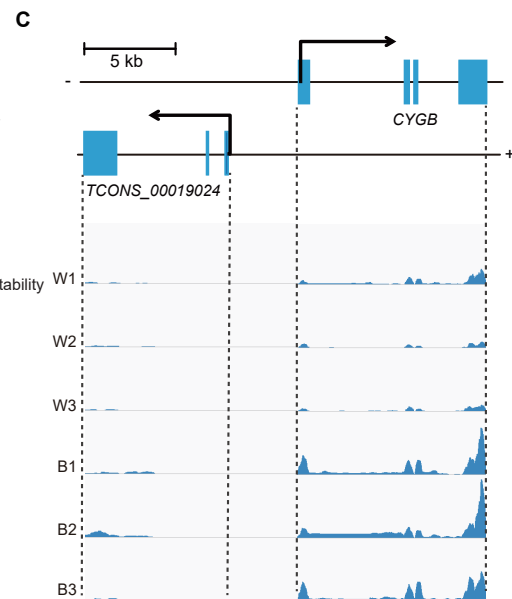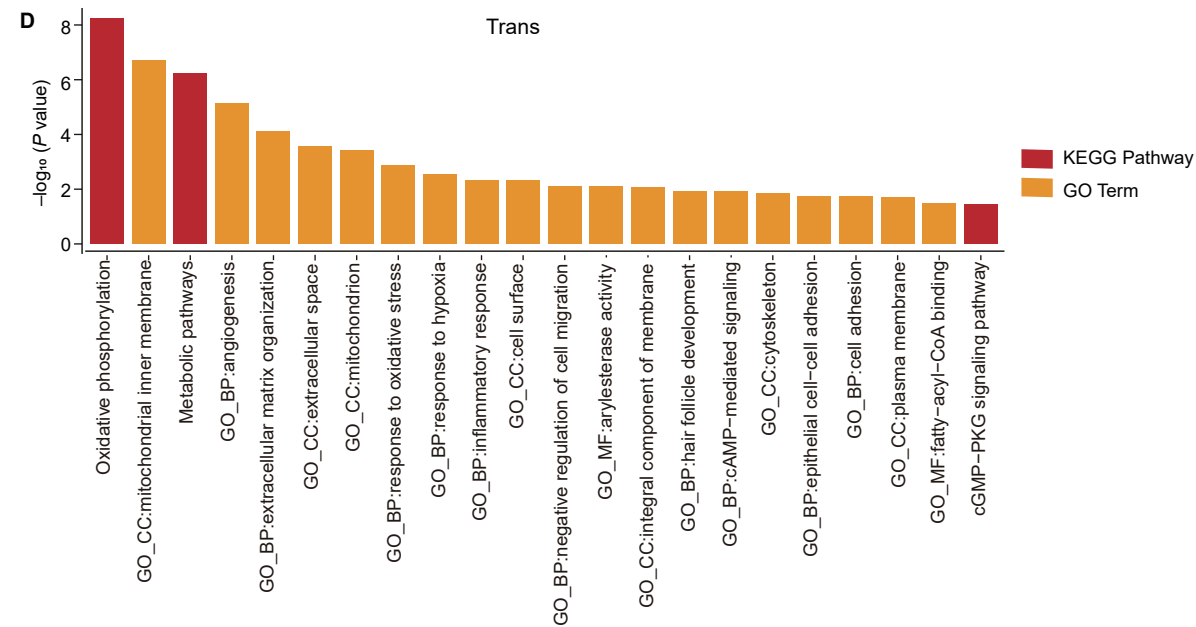

Supplement: Supplementary file 1 [file genes-11-00047-s001.zip › Figure.4.pdf]

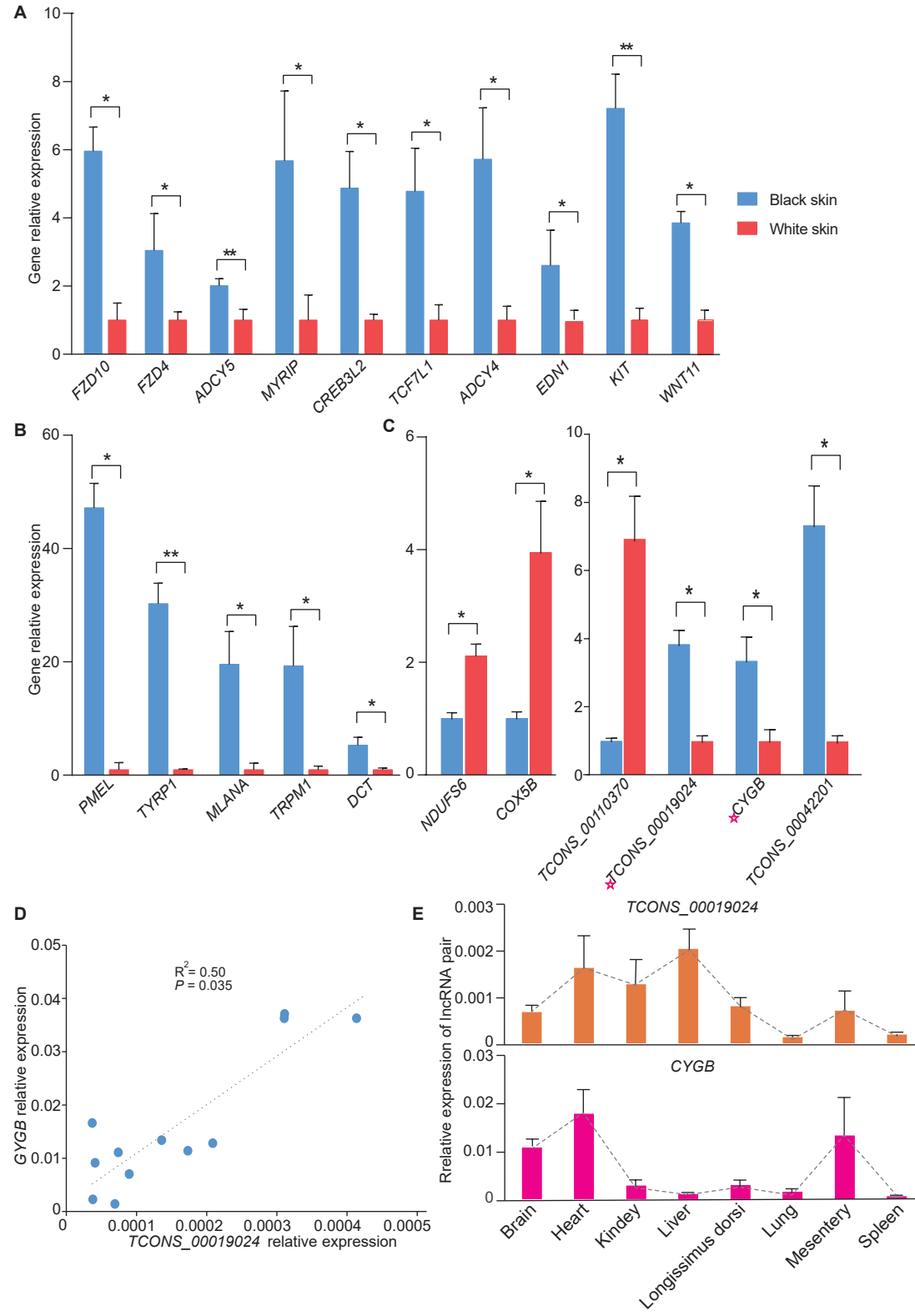

Supplement: Supplementary file 1 [file genes-11-00047-s001.zip › Figure.5.pdf]

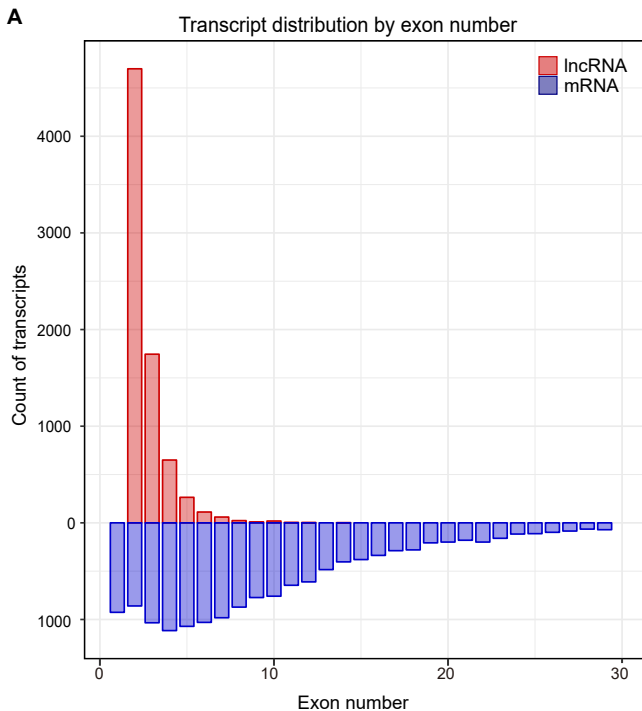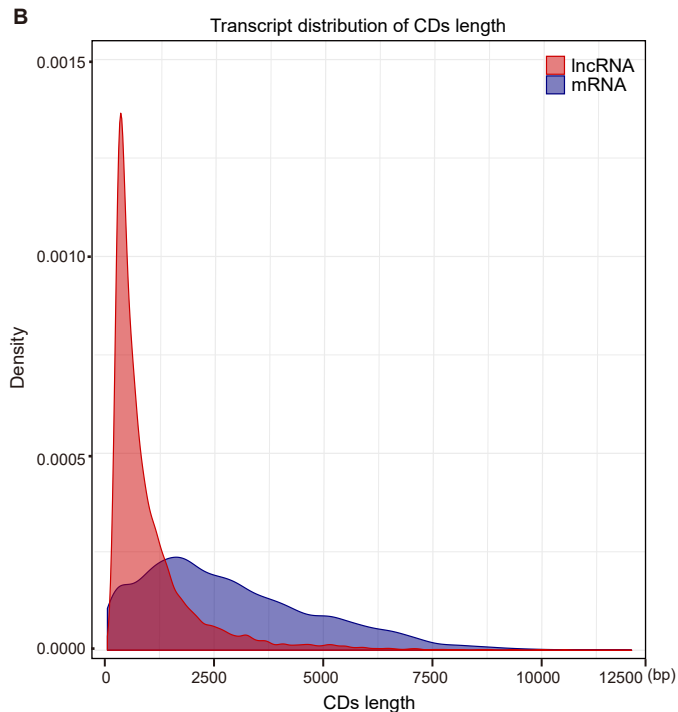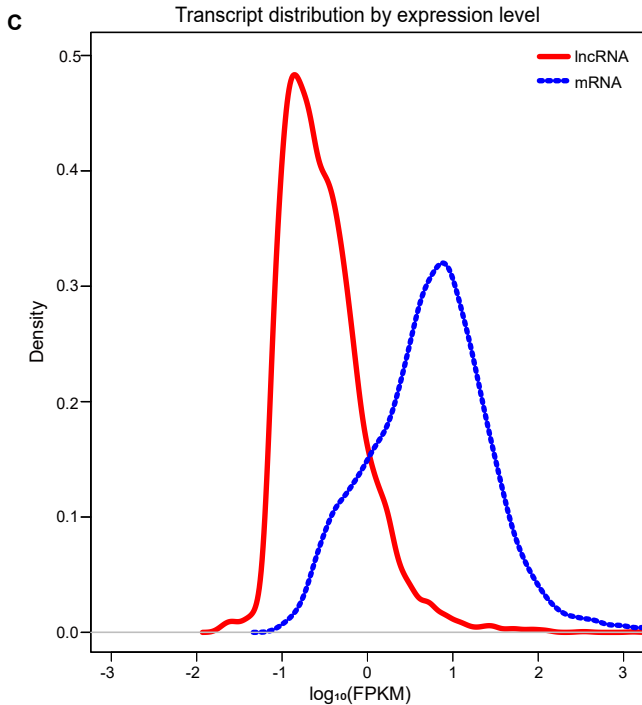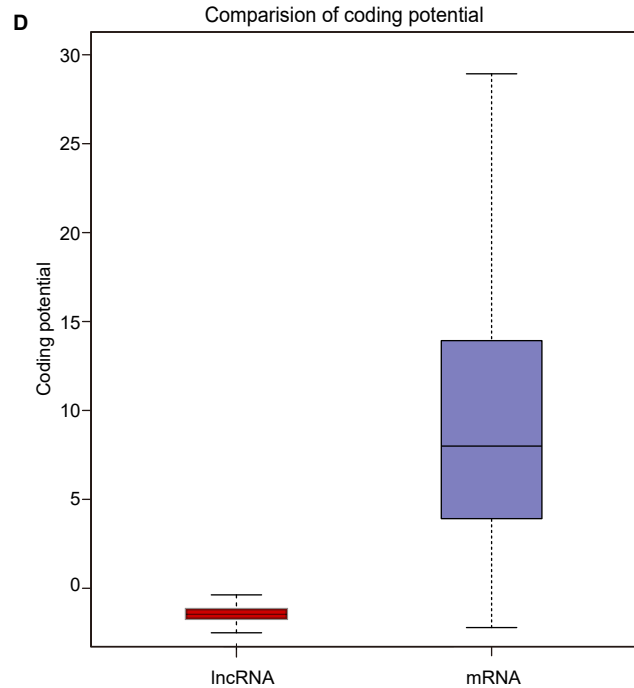

Supplement: Supplementary file 1 [file genes-11-00047-s001.zip › Figure.S1.pdf]

**A**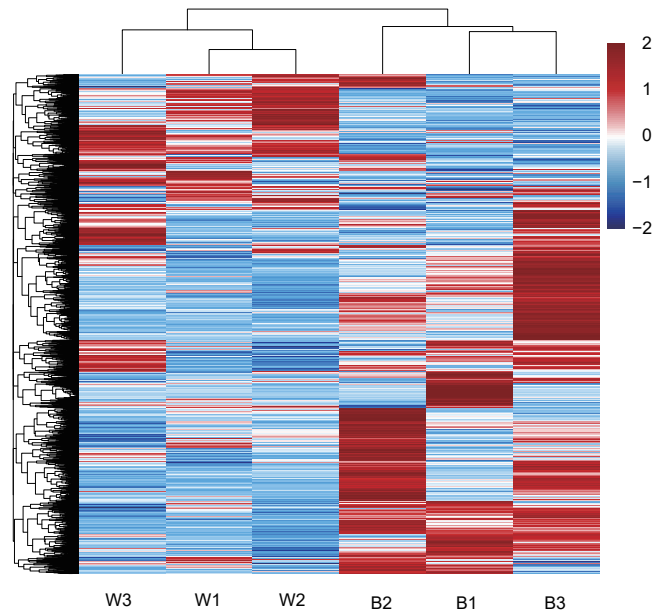**B**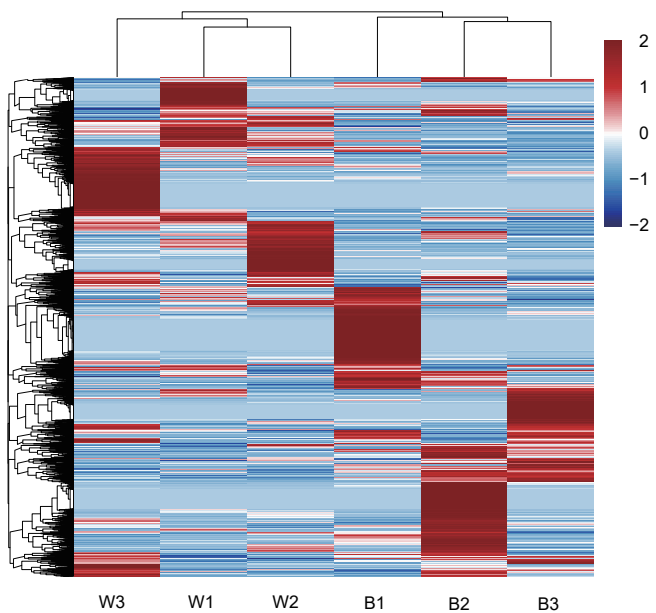**C**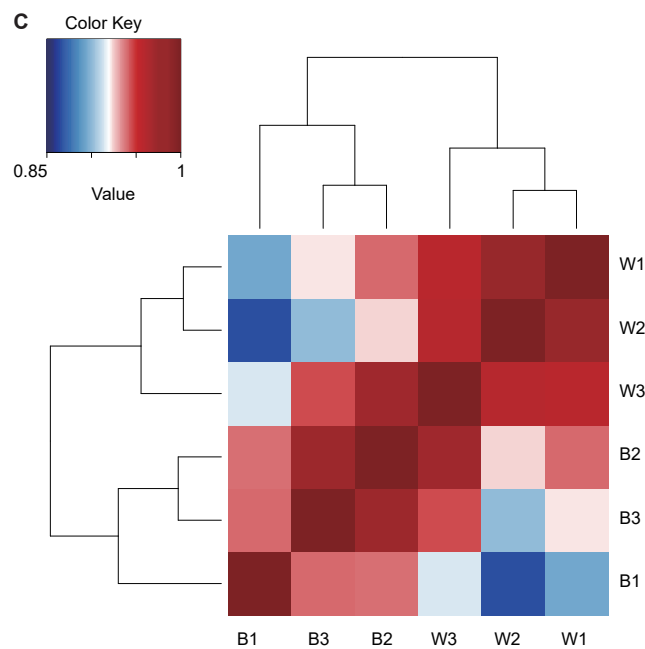**D**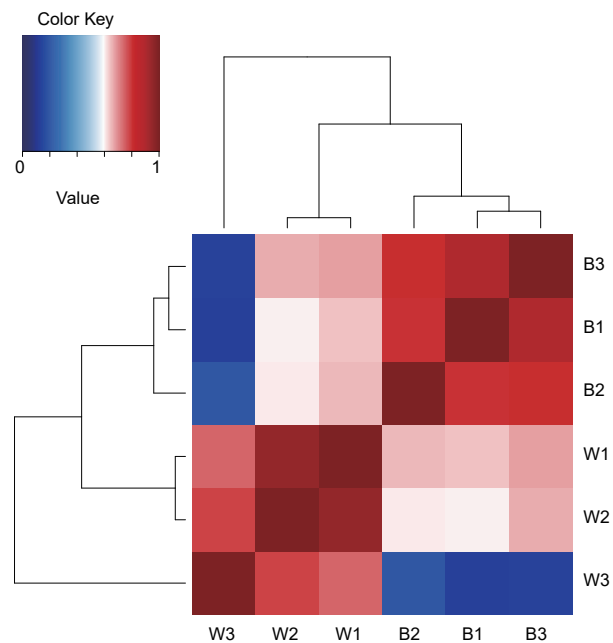

Supplement: Supplementary file 1 [file genes-11-00047-s001.zip › Figure.S2.pdf]

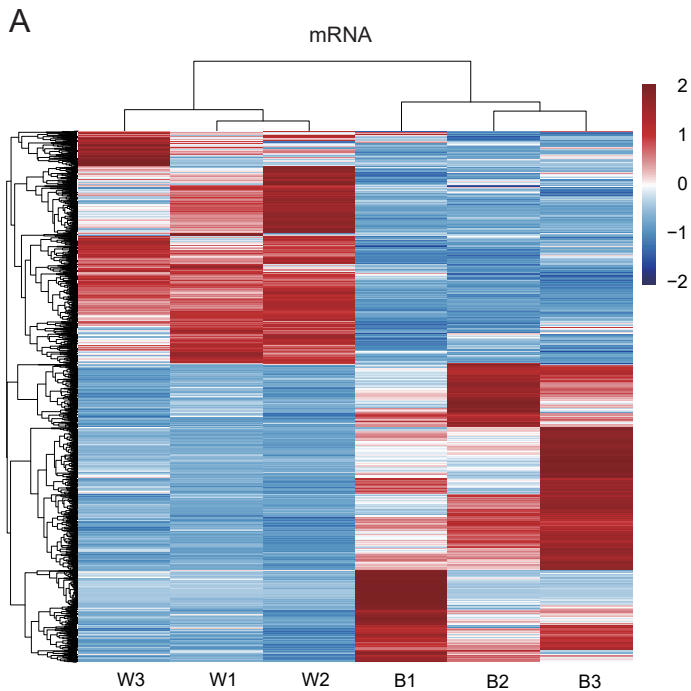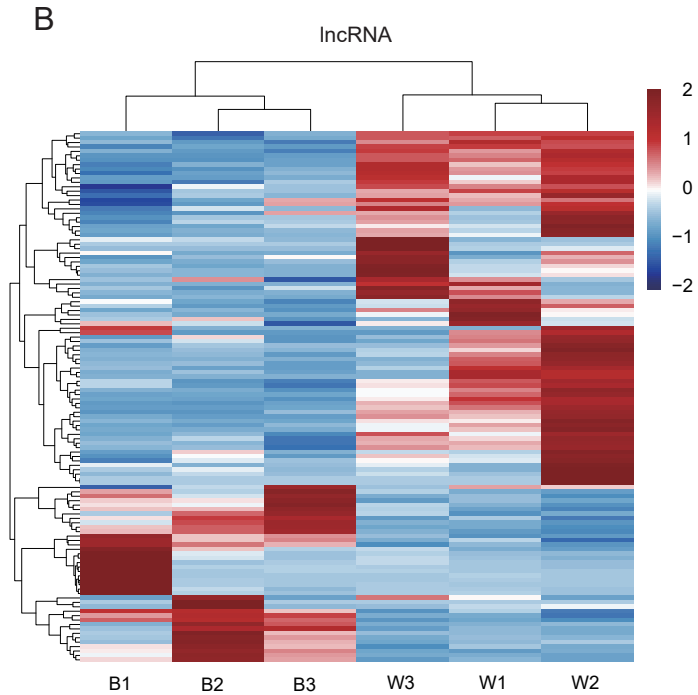

Supplement: Supplementary file 1 [file genes-11-00047-s001.zip › Figure.S3.pdf]

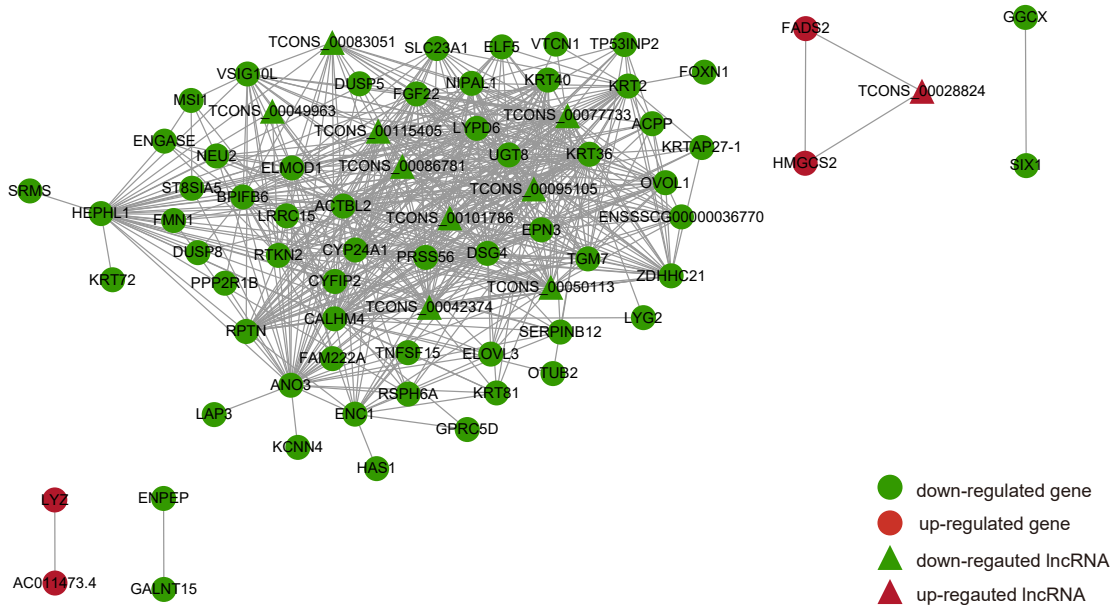

Supplement: Supplementary file 1 [file genes-11-00047-s001.zip › Figure.S4.pdf]
